# How DNA barcoding can be more effective in microalgae identification: a case of cryptic diversity revelation in *Scenedesmus* (Chlorophyceae)

**DOI:** 10.1038/srep36822

**Published:** 2016-11-09

**Authors:** Shanmei Zou, Cong Fei, Chun Wang, Zhan Gao, Yachao Bao, Meilin He, Changhai Wang

**Affiliations:** 1 College of Resources and Environmental Science, Nanjing Agricultural University, Nanjing 210095, PR China

## Abstract

Microalgae identification is extremely difficult. The efficiency of DNA barcoding in microalgae identification involves ideal gene markers and approaches employed, which however, is still under the way. Although *Scenedesmus* has obtained much research in producing lipids its identification is difficult. Here we present a comprehensive coalescent, distance and character-based DNA barcoding for 118 *Scenedesmus* strains based on *rbcL*, *tufA*, ITS and 16S. The four genes, and their combined data *rbcL* + *tufA* + ITS + 16S, *rbcL* + *tufA* and ITS + 16S were analyzed by all of GMYC, P ID, PTP, ABGD, and character-based barcoding respectively. It was apparent that the three combined gene data showed a higher proportion of resolution success than the single gene. In comparison, the GMYC and PTP analysis produced more taxonomic lineages. The ABGD generated various resolution in discrimination among the single and combined data. The character-based barcoding was proved to be the most effective approach for species discrimination in both single and combined data which produced consistent species identification. All the integrated results recovered 11 species, five out of which were revealed as potential cryptic species. We suggest that the character-based DNA barcoding together with other approaches based on multiple genes and their combined data could be more effective in microalgae diversity revelation.

Microalgae are diverse and ubiquitous in aquatic and some terrestrial habitats. They play a crucial role in the global ecosystem for hundreds of millions of years^1^,^2^. The revelation of biodiversity for microalgae is significant to nature conversation, food safety and better understanding the patterns of ecosystem functioning. The Chlorophyta form a large and morphologically diverse clade of marine, freshwater and terrestrial green algae. Although Chlorophyta have a long history of study, they are still poorly understood taxonomically and phylogenetically due to their much diversity, especially for microalgae. Microscopic green algae are mainly identified based on the general shape of their vegetative cells, the position of chloroplasts and pyrenoids, and the ultrastructural characteristics^3^,^4^. However, identification of microalgae can be very difficult since most species lack obvious structural features and some of the observable characteristics are variable within species. Since the identification of microalgae typically requires the use of a microscope, sometimes at very high magnification, taxonomy of it is somewhat inaccessible to non-specialists and sometimes rapid identification of some species even by microscopy is impossible. Even worth, the number of taxonomists is declining seriously.

The genus *Scenedesmus* (Chlorophyceae) is one of the most common freshwater genera, which can be as ideal microalgae for producing biofuel owing to the substantial amounts of lipids, proteins and carbohydrates. Most species of *Scenedesmus* are found across the world. *Scenedesmus* includes all autosporic coccal green algae with flat or curved coenobia^5^, species of which are poor in characteristics and are differentiated mainly by cell shape or coenobial habitus^6^. The extremely diverse morphologies make identification of *Scenedesmus* very difficult^7^. Currently, there are 74 taxonomically accepted species of *Scenedesmus*^8^, but not determinate. It is hard to distinguish them just based on the limited obscure characters. At present, there are few studies about taxonomic assignments of *Scenedesmus* using molecular tools^5^,^9^, and these studies just used single gene or limited analysis. It is urgent to give a revision to the classification of *Scenedesmus*.

DNA barcoding is currently a widely used and effective tool for fast and accurate species identification^10^,^11^,^12^,^13^,^14^,^15^. The efficient “universal barcode gene” across all forms of life is a key factor for success application of DNA barcoding. However, the weakest spot of DNA barcoding is the obvious fact that no gene can serve as an ideal barcode for all forms of life, i.e., be always invariant within species but different among species. While the application of cytochromeoxidase- I (COI) has been highly successful in a wide range of animal taxa^16^, the attempts to employ a single barcode for plants identification remains a vain hope for a longtime. The cpDNA two-locus combination *rbcL* + *matK* has been recommended as the universal DNA barcode for land plants^17^. However, the discriminatory power of the *rbcL* + *matK* sequence combination is still very far from the usually higher (though variable) rate of over 90% success of COI in animals^16^,^18^. Moreover, the *matk* is absent in algae. Thus, the efficient “DNA barcodes” for algae are more ambiguous, and it seems more effective to employ multiple genes for barcoding algae. Several gene loci, e.g. *rbcL*, ITS and *tufA*, have been recommend as the promising DNA barcodes for some green algae^19^,^20^.

Members of the barcoding community have put forward several different methods of distinguishing species, including the coalescent, distance and character-based methods. Traditional DNA barcoding^10^ constructs Neighbor-Joining (NJ), Bayesian or Maximum likelihood trees for species identification, and calculates a genetic distance between species and assigns a cutoff value (the ‘barcode gap’) to divide OTUs into species. The distinct clades in a phylogenetic tree are often interpreted as species. However, monophyly of a set of taxa can occur by chance within a larger panmictic group as a result of the coalescent process. Recently, the P ID(Liberal) method of species delimitation is advanced for the exploration of species boundaries which are identified by deep divergences in phylogenetic trees^21^. Another popular tree-based approach, the General Mixed Yule-coalescent^22^,^23^, is a species delimitation method that estimates species boundary directly from branching rates in a phylogenic tree rather than actual sequence data and attempts to statistically model the point on a time calibrated (ultrametric) phylogeny. The single-threshold approach is generally preferred for GMYC analysis^24^. The poisson tree process (PTP) model is another tree-based method that distinguishes specimens in both populations and species level using coalescence theory^25^. It has been proposed that P ID, GMYC and PTP approaches could be as complementary analysis to the phylogenetic tree identification of traditional DNA barcoding^26^,^27^,^28^. For distance-based barcoding approach, due to the absence of a “barcoding gap”, the specimen identification based on intraspecific variation vs. interspecific divergence has already been shown to be impossible for some taxonomic groups, especially for the plants^29^,^30^,^31^. Recently, a new distance method, called Automatic Barcode Gap Discovery (ABGD), is proposed as an automatic procedure that sorts the sequences into hypothetical species based on the barcode gap which can be observed whenever the divergence among organisms belonging to the same species is smaller than divergence among organisms from different species^32^. Another new barcoding method, the character-based barcoding which is different from phylogenetic tree and distance analysis, is based on the fundamental concept that members of a given taxonomic group share diagnostic characters that are absent from comparable groups^33^,^34^. It can provide better resolution in species identification and cryptic species revelation of some organisms (including some plants) in several cases where distance-based methods fail to distinguish species^12^,^31^.

Here we present the comprehensive DNA barcoding taxonomic assignment of *Scenedesmus* based on four gene loci and their combined data, the *rbcL* gene (encodes the large subunit of Rubisco), the *tufA* gene (encoding elongation factor), the ITS (internal transcribed spacer region) and 16S. This study represents one of the efforts to use DNA barcode data as a taxonomic tool for exploring biodiversity of microalgae. We employ a novel combination of methods to reach this goal, examining the congruence of OTUs (operational taxonomic units) resulting from coalescent (P ID, GMYC and PTP), distance (ABGD) and character-based DNA barcoding. The objectives of this study are: (1) to identify the confused *Scenedesmus* strains; (2) to uncover the cryptic species in *Scenedesmus*; (3) to test the efficiency of multiple gene markers and barcoding approaches; (4) to evaluate how DNA barcoding can be more effective in microalgae diversity revelation.

## Results

**Figure 1:**
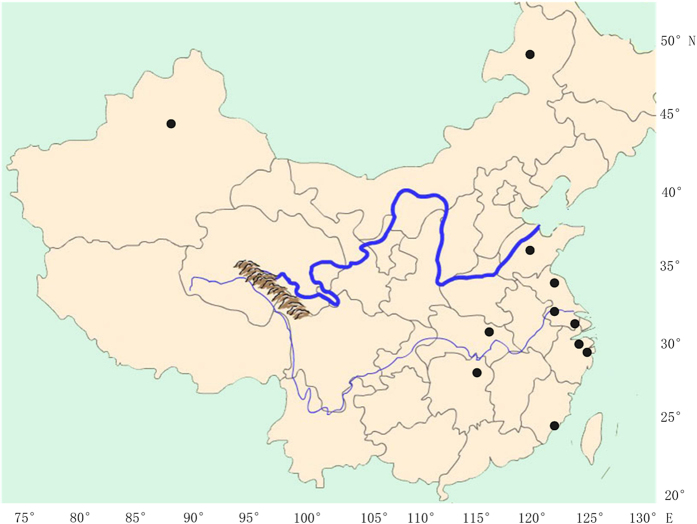
Map showing the locations from which *Scenedesmus* strains were obtained from China (shown as dark dots). The map was created using Quantum GIS 1.8.0 ( http://www.qgis.org/) based on a map from Natural Earth (version 2.0.0).

A total of 68 *rbcL*, 80 ITS, 64 16S and 54 *tufA* sequences of *Scenedesmus* samples and outgroups were analyzed (Supplementary Table 1). The samples from this study were selected from many regions of China (Fig. 1). The newly obtained sequences from this study were submitted to the GenBank Barcode database with accession numbers KT777944- KT778122 and KT818697- KT818720. The *rbcL* sequence had a length of 1323 bp with 740 variable nucleotide sites (55.9%), 605 of which were non-synonymous substitutions. The ITS sequence had a length of 1354 bp with 582 variable nucleotide sites (43.0%), 514 of which were non-synonymous substitutions. The 16S sequence had a length of 436 bp with 209 variable nucleotide sites (48.0%), 179 of which were non-synonymous substitutions. The *tufA* sequence had a length of 789 bp with 459 variable nucleotide sites (58.2%), 393 of which were non-synonymous substitutions.

### Single marker barcoding

**Figure 2:**
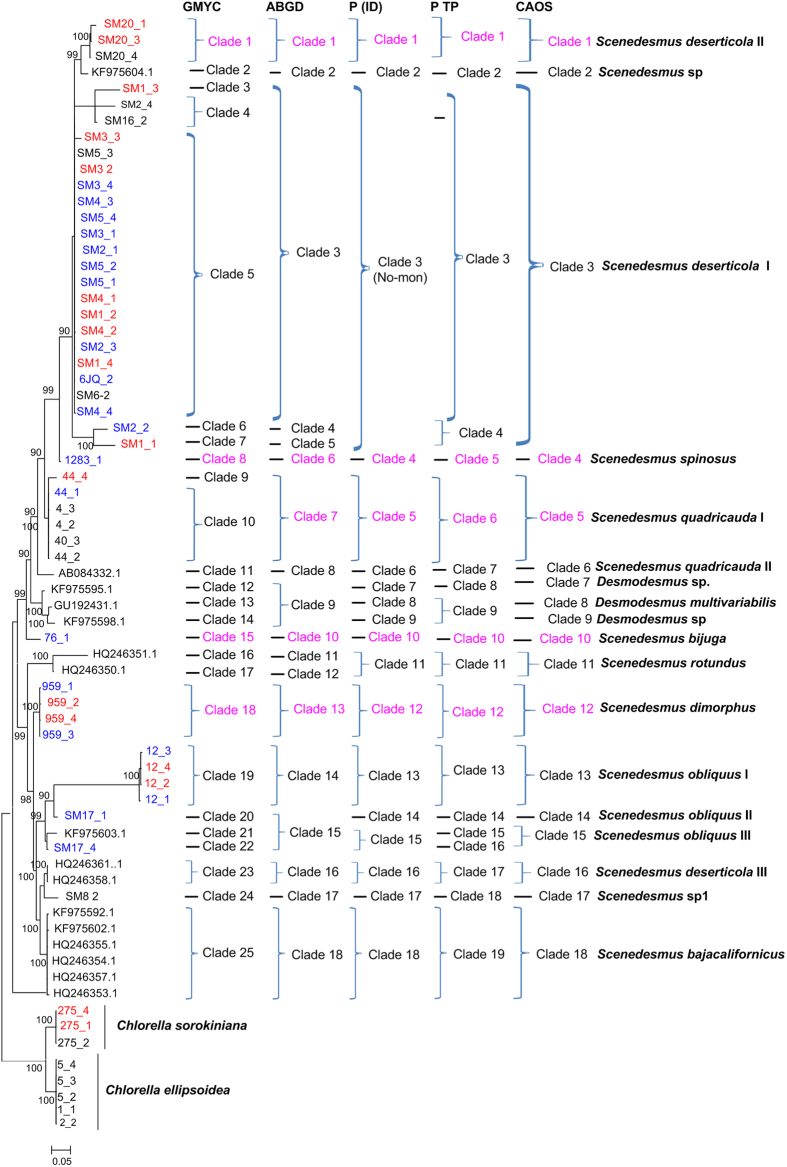
Bayesian phylogenetic tree for the *rbcL* gene. Vertical brace on the right indicate the clades detected by the tree-based GMYC, PID, PTP and the distance-based ABGD approach, the character-based CAOS and the taxa assignment. The clades highlighted in pink were also detected by 16S, ITS and *tufA* gene loci. For samples colored in red, 16S, ITS and *tufA* sequences were also available. For specimens colored in blue, two of 16S, ITS and *tufA* sequences were available.

Generally, the NJ, Bayesian and Maximum Likelihood trees of *rbcL* recovered consistent groups (Fig. 2, Supplementary Fig. 1 and Supplementary Fig. 2), including the potential cryptic lineages (e.g *Scenedesmus deserticola* I,II,III and *Scenedesmus obliquus* I,II,III). As can be seen in Supplementary Fig. 3, Supplementary Fig. 4, Supplementary Fig. 5, Supplementary Fig. 6, Supplementary Fig. 7, Supplementary Fig. 8, Supplementary Fig. 9, Supplementary Fig. 10 and Supplementary Fig. 11, the NJ, Bayesian and Maximum Likelihood trees of *tufA*, ITS and 16S also retrieved generally consistent groups. However, it could also be seen that some clades could not be separated clearly in the *rbcL*, ITS, 16S and *tufA* phylogenetic trees, e.g. the *S. deserticola* I and *S. deserticola* II clades.

The distance variation of *rbcL*, ITS, *tufA* and 16S among taxa assignments by P ID, ABGD, GMYC, PTP and CAOS analysis were conducted. The results showed that the mean intraspecific distance of *rbcL* was from 0% to 1.64% while the mean interspecific distances was from 0.4% to 23.0% (Supplementary Table 2). All the pairwise distance of *rbcL* ranged from 0% to 32.5%. The mean intraspecific distance of ITS ranged from 0% to 4.08% while the mean interspecific distances was from 1.1% to 21.70% (Supplementary Table 3). All the pairwise distance of ITS ranged from 0% to 24.3%. For 16S and *tufA*, the mean intraspecific distance ranged from 0% to 2.84% and 0% to 4.54% respectively while the mean interspecific distances was from 0% to 35.1% and 1.9% to 38.0% respectively (Supplementary Table 4 and Supplementary Table 5). The pairwise distance of 16S and *tufA* ranged from 0% to 36.7% and 0% to 38.0% respectively. All these distance variation showed that a DNA “barcoding gap” did not existed in *rbcL*, 16S, ITS and *tufA* sequences.

Based on the distance-based approach (‘Barcode-gap analysis’) as implemented in the software ABGD, different groups as candidate species were produced for *rbcL*, ITS, 16S and *tufA* gene sequences. The ABGD analysis of *rbcL*, ITS, 16S and *tufA* did not always produce consistent genetic groups for all species (Fig. 2, Supplementary Fig. 3, Supplementary Fig. 6 and Supplementary Fig. 9). For *rbcL*, the ABGD analysis revealed 18 genetic groups when using restrictive values with priori genetic distance thresholds between 0.46 and 0.77% (Fig. 2 and Supplementary Fig. 12). This result was generally consistent with the ABGD analysis of ITS in which 16 groups were produced with prior maximal distance 1.29% (Supplementary Fig. 6 and Supplementary Fig. 13). In both analyses, *S. deserticola* was split into several groups. All the specimens studied were split into more groups in ABGD analysis of 16S where 21 groups were revealed with priori genetic distance thresholds between 0.28 and 0.46% (Supplementary Fig. 9 and Supplementary Fig. 14). However, the ABGD analysis of *tufA* could not separate most specimens in which only 10 groups were produced with priori genetic distance thresholds between 0.28 and 2.15% (Supplementary Fig. 3 and Supplementary Fig. 15).

Based on the Bayesian analysis, the tree-based hypotheses were reevaluated for species hypothesis testing by P ID species boundary delimitation. Most candidate species were recovered as monophyletic clades in all of *rbcL*, ITS, *tufA* and 16S genes except *S. deserticola* I which was not monophyletic in P ID analysis of *rbcL*, ITS and 16S (Fig. 2, Supplementary Fig. 3, Supplementary Fig. 6 and Supplementary Fig. 9). Several cryptic clades of *S. obliquus* were also not monophyletic in 16S P ID analysis. All delimited species of *rbcL*, ITS, *tufA* and 16S sequences possessed a P ID (Liberal) value P > 0.5 except two clades in 16S analysis (Supplementary Table 6, Supplementary Table 7, Supplementary Table 8 and Supplementary Table 9).

On the whole, the specimens analyzed were oversplitted by the GMYC model in comparison with the ABGD and P ID analysis in *rbcL*, ITS and *tufA* genes (Fig. 2, Supplementary Fig. 3, Supplementary Fig. 6, Supplementary Fig. 9, Supplementary Fig. 16, Supplementary Fig. 17 and Supplementary Fig. 18). The results of single threshold analysis for the *rbcL*, ITS and *tufA* gene suggested 25, 25 and 19 groups respectively. In 16S gene dataset ten GMYC entities recovered was generally congruent with the P ID species boundary delimitation Supplementary Fig. 19.

For bPTP approach, the maximum-likelihood identification was showed since it produced better resolution than bayesian identification. The taxonomic clades produced by bPTP approach was variable among *rbcL*, ITS, *tufA* and 16S genes (Fig. 2, Supplementary Fig. 6, Supplementary Fig. 20, Supplementary Fig. 21, Supplementary Fig. 22 and Supplementary Fig. 23). It was apparent that the *rbcL* and ITS genes generated better identification than that of *tufA* and 16S genes. The *tufA* and 16S genes did not distinguish most species (Supplementary Fig. 22 and Supplementary Fig. 23). For *rbcL*, it recognized 19 independent entities, some of which were consistent with the groups revealed by GMYC, ABGD or P ID analyses (Fig. 2). For ITS, 11 clades were identified by PTP analysis, which were not completely consistent with the identification of other methods (Supplementary Fig. 6 and Supplementary Fig. 21).

The relatively congruent defined *Scenedesmus* groups based on ABGD, P ID, PTP and GMYC analysis, as well as the morphological characters, were analyzed for searching for diagnostic characters. A total of 18, 14, 14 and 15 clades recovered by *rbcL*, ITS, *tufA* and 16S genes were analyzed respectively by character-based DNA barcoding (Fig. 2, Supplementary Fig. 3, Supplementary Fig. 6 and Supplementary Fig. 9). It was found that all the *Scenedesmus* groups including the possible cryptic lineages and unknowns were clearly distinguished in the character-based DNA barcoding. In the *rbcL* gene region of 18 *Scenedesmus* taxa recovered in Fig. 2, character states at 27 nucleotide positions were detected (Table 1). All the 18 clades revealed a unique combination of character states at 27 nucleotide positions with more than three CAs, including the cryptic lineages, unknowns and species represent by single specimens. As can be seen in Supplementary Table 10, 14 clades recovered in Supplementary Fig. 6, also revealed a unique combination of character states at 28 nucleotide positions with more than three CAs, for ITS sequences. The 16S character states for the 15 *Scenedesmus* clades (Supplementary Fig. 9) were shown in Supplementary Table 11. At 25 nucleotide positions of the 16S gene region more than four CAs were revealed for each clade. The *tufA* character-based DNA barcode were shown in Supplementary Table 12, in which 14 defined *Scenedesmus* clades recovered in Supplementary Fig. 3 revealed a unique combination of character states at 39 positions.

### Comparison of species delimitation by traditional DNA barcoding, ABGD, P ID, GMYC, PTP and character-based methods in four gene loci

As seen in Fig. 2, Supplementary Fig. 3, Supplementary Fig. 6 and Supplementary Fig. 9, relatively congruent clades were recovered by *rbcL*, ITS, 16S and *tufA* genes. For example, the species *Scenedesmus deserticola* II, *Scenedesmus deserticola* I, *Scenedesmus bijuga, Scenedesmus dimorphus*, *Scenedesmus quadricauda* and *Scenedesmus bajacalifornicus* were recovered as monophyletic clades in all of *rbcL*, ITS, 16S and *tufA* genes by more than three methods of Bayesian trees, ABGD, P ID, GMYC, PTP and character-based. However, on the other hand, the Bayesian trees, ABGD, P ID, GMYC, PTP and character-based methods did not always generate consistent clades in each of *rbcL*, ITS, 16S and *tufA* genes. As a whole, the GMYC method produced more OTUs than other methods. The PTP approach just could identify most species in *tufA* and ITS genes. It was apparent that the character-based method recovered consistent clades among *rbcL*, ITS, 16S and *tufA* gene loci. In comparison with *rbcL*, 16S and ITS, the *tufA* showed a higher intraspecific and interspecific divergence, more consistent groups among GMYC, ABGD and P ID methods, and more diagnostic characters.

### Combined markers barcoding

**Figure 3:**
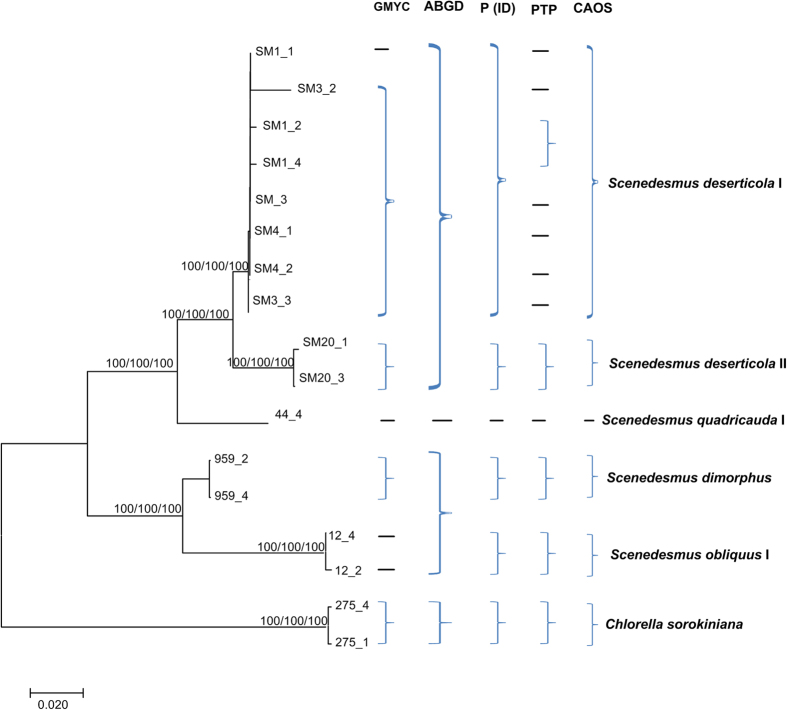
Bayesian phylogenetic tree for the *rbcL* + ITS + 16S + *tufA* data. The NJ and Maximum Likelihood bootstrap were also indicated. Vertical brace on the right indicate the clades detected by the tree-based GMYC, PID, PTP, the distance-based ABGD approach and the character-based CAOS assignment.

The combined data of *rbcL* + ITS + 16S + *tufA*, *rbcL* + *tufA* and ITS + 16S were analyzed respectively, based on the ABGD, P ID, GMYC, PTP and character-based methods. The *rbcL*, ITS, 16S and *tufA* sequences were all available for *S. deserticola* I, *S. deserticola* II, *S. quadricauda*, *S. obliquus*, and *S. dimorphus* (Fig. 3). The NJ, Bayesian and Maximum Likelihood trees of *rbcL* + ITS + 16S + *tufA* separated the five clades clearly, with high support. The ABGD analysis revealed 4 genetic groups when using restrictive values with priori genetic distance thresholds between 2.15% (Fig. 3 and Supplementary Fig. 24), which did not distinguish *Scenedesmus deserticola* I and *Scenedesmus deserticola* II, and *S. obliquus*, and *S. dimorphus*. The five species were divided into 8 genetic groups by the GMYC model (Supplementary Fig. 25), which was consistent with the single gene result that GMYC model could separate one species as more clades. P ID species boundary delimitation revealed all the several species as monophyletic clades. The delimited species of *rbcL* + ITS + 16S + *tufA* sequences possessed a P ID (Liberal) value P>0.9 (Supplementary Table 13). As the GMYC analysis, the PTP approach also divided the *Scenedesmus deserticola* I into several clades (Fig. 3 and Supplementary Fig. 26). The character-based barcoding separated the five species more clearly (Table 2). It was indicated that every species revealed in Table 2 possessed more than 7 character attributes in only 19 positions.

**Figure 4:**
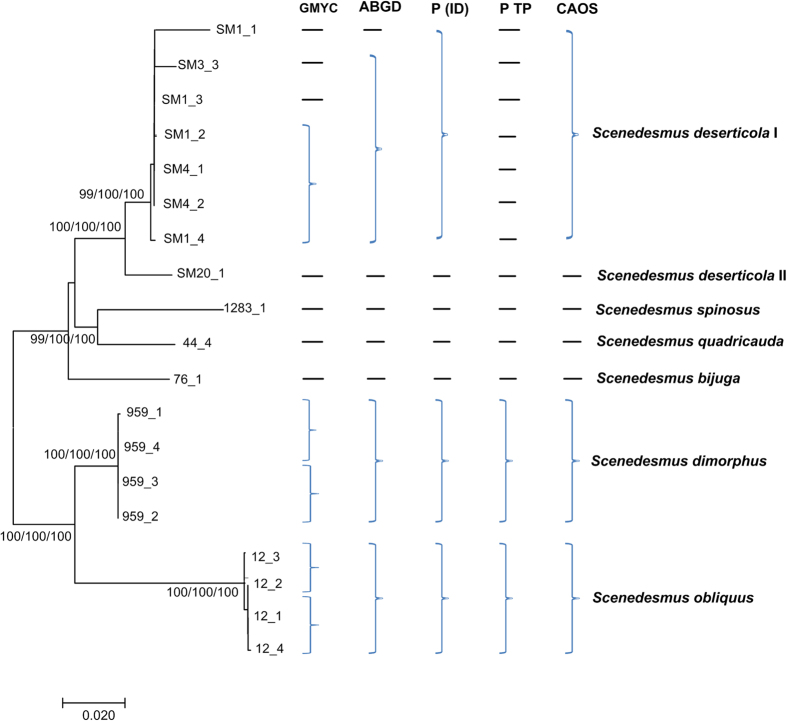
Bayesian phylogenetic tree for the *rbcL* + *tufA* data. The NJ and Maximum Likelihood bootstrap were also indicated. Vertical brace on the right indicate the clades detected by the tree-based GMYC, PID, PTP, the distance-based ABGD approach and the character-based CAOS assignment.

The *rbcL* + *tufA* sequences revealed similar resolution to *rbcL* + ITS + 16S + *tufA*. Both *rbcL* and *tufA* sequences were all available for 7 species (Fig. 4). The 7 clades were also clearly separated by NJ, Bayesian and Maximum Likelihood analysis of *rbcL* + *tufA* with strong support. The ABGD analysis revealed 8 genetic groups when using restrictive values with priori genetic distance thresholds between 0.1–0.45% (Fig. 4 and Supplementary Fig. 27). The 7 species were also oversplitted by the GMYC model (Supplementary Fig. 28). All the several species were also revealed as monophyletic clades by P ID species boundary delimitation, and the delimited species of *rbcL* + *tufA* sequences possessed a P ID (Liberal) value P > 0.8 (Supplementary Table 14). It was apparent that the PTP method divided *S. deserticola* I into more separate clades (Supplementary Fig. 29). As *rbcL* + ITS + 16S + *tufA* recovered, the character-based barcoding also separated the seven species clearly (Table 3). Seven defined *Scenedesmus* clades recovered in Fig. 4 revealed a unique combination of character states at 29 positions with more than 8 character attributes.

**Figure 5:**
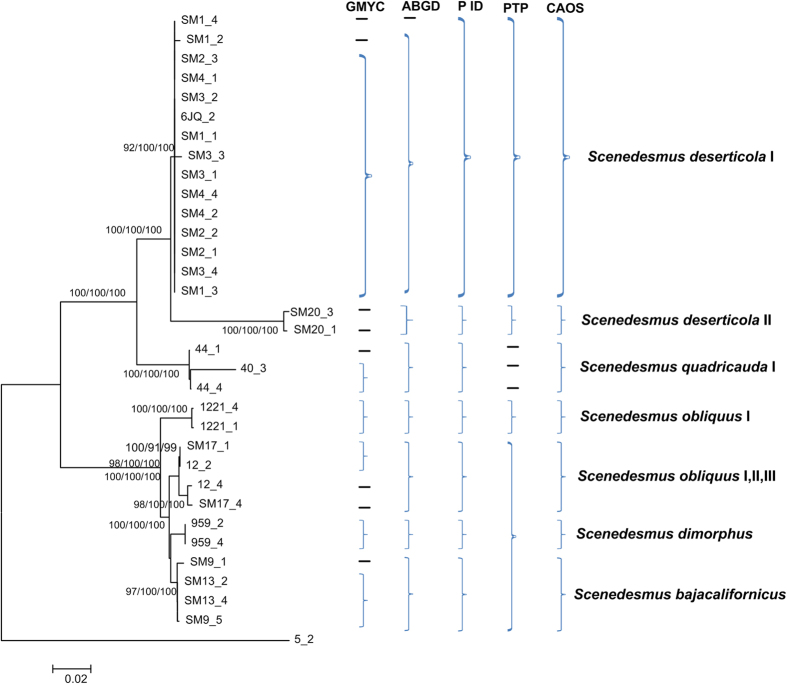
Bayesian phylogenetic tree for the ITS + 16S data. The NJ and Maximum Likelihood bootstrap were also indicated. Vertical brace on the right indicate the clades detected by the tree-based GMYC, PID, PTP, the distance-based ABGD approach and the character-based CAOS assignment.

The identification of ITS + 16S sequences by GMYC, P ID, PTP, ABGD and character-based methods were showed in Fig. 5. It was indicated that generally the ABGD, P ID and character-based barcoding recovered same resolution where seven genetic lineages were clearly separated, including the potential cryptic lineages *S. deserticola* and *S. obliquus.* The ABGD analysis revealed 8 genetic groups. The P ID species boundary delimitation of ITS + 16S sequences revealed all species as monophyletic clades, and the delimited species possessed a P ID (Liberal) value P > 0.6 (Supplementary Table 15). Both the GMYC model and PTP analysis generated more genetic lineages compared with the ABGD, P ID and the character-based methods (Fig. 5, Supplementary Fig. 30 and Supplementary Fig. 31). The character-based barcoding separated the seven taxonomic lineages clearly (Fig. 5, Table 4). It was indicated that every clade revealed in Table 4 possessed more than 5 character attributes in only 15 positions.

## Discussion

Identification of microalgae species is often difficult based on the morphological characters due to their tiny body, unobvious structural features and variable characters within species. DNA barcoding has developed to be a useful tool for species discrimination. However, how DNA barcoding can be more effective in microalgae diversity revelation by selecting suitable markers and barcoding approaches is ambiguous. The identification of *Scenedesmus* by morphological characters is often confused, which hinders us from selecting optimal *Scenedesmus* strains for producing biofuel. In this study, the morphological characters were initially used to identify the strains. Some strains could easily be identified to species level. However, although some strains could be identified to species level they seemed to be potential cryptic species due to the various morphological characters. Here we employ multiple genes to assign *Scenedesmus* species based on various barcoding methods, and evaluate their congruent results.

Generally, different gene markers produced consistent resolution for species discrimination. Most species, including the unknowns, were separated clearly in barcoding analysis of *rbcL*, ITS, 16S, *tufA* and the three combined data. For example, the species *S. bajacalifornicus*, *S. dimorphus* and *S. quadricauda* were all clearly distinguished from other species by P ID, ABGD, GMYC and CAOS analysis of *rbcL*, ITS and 16S sequences, or the three combined data. More importantly, some species which were identified as potential cryptic species complexes were also divided into several separate lineages by the gene sequences. As can be seen in Figs 2, 3, 4 and 5, and Supplementary Fig. 3, Supplementary Fig. 6 and Supplementary Fig. 9, *S. deserticola* were clearly retrieved as separate clades in all barcoding analysis of *rbcL*, ITS, 16S, *tufA* and the three combined data, including the sequences from Genbank. Additionally, *S. obliquus*, *S. quadricauda*, *S. spinosus* and *S. acuminatus* were also recovered as separate clades. These species might be as overlooked cryptic species. Since *S. deserticola*, *S. obliquus*, *S. quadricauda* and *S. acuminatus* are all considered to be promising candidates for biodiesel production^35^,^36^ their correct identification is significant to the application as biodiesel feedstock. In sum, the molecular study of DNA barcoding here gave new insights into the taxonomic assignment of *Scenedesmus.*

Since mitochondrial genes are not suitable for barcoding plantae^37^ the chloroplast and nuclear genomes with high substitution rates could be employed to search for plant barcodes. Although *rbcL* + *matK* are proposed as candidates of DNA barcode loci for plants it has been proved that the *matK* or *rbcL* alone can not be as a suitable universal barcode^37^,^38^. In the present study, the four gene loci and the combined data generally produced congruent clades among Bayesian, ABGD, GMYC, P ID, PTP and character-based analysis, respectively. By comparison, all of the four-marker combination of *rbcL* + ITS + 16S + *tufA* and two-marker combination of *rbcL* + *tufA* and ITS + 16S showed a much higher proportion of resolution success than the single genes, including the more consistent groups among GMYC, ABGD, PTP, and P ID analysis, and many more diagnostic characters. Among the four genes, the *tufA* generally produced better resolution than other genes, also including the higher intraspecific and interspecific divergence, more consistent groups among GMYC, ABGD and P ID analysis, and many more diagnostic characters.

This study represents one of the first efforts to examine the congruence of barcoding results from multiple delimitation methods. The P ID, PTP and Character methods were particularly included in this study in comparison with previous barcoding evaluation^26^,^27^. The traditional barcoding analysis, including the phylogenetic (NJ, Bayesian and Maximum Likelihood analysis) and intra and interspecific distance analysis, was first conducted to discriminate species. Due to the drawback of monophyly-based species identification^39^,^40^ it is more likely the phylogenetic trees could be used as the initial step to identify putative independently-evolving lineages. It has been proposed that an optimal path to understand species boundaries is starting with a tree-based framework to develop the initial species hypotheses where distinct clades can be identified as divergent monophyletic population clusters^27^. In this study, as a whole, the NJ, Bayesian and Maximum Likelihood phylogenetic trees produced consistent topology for each marker of *rbcL*, ITS, 16S, *tufA*, *rbcL* + *ITS* + *16S* + *tufA*, *rbcL* + *tufA* and ITS + 16S. For all of *rbcL*, ITS, 16S and *tufA* sequences, although the interspecific distance was generally higher than the intraspecific distance, there was no barccoding gap between them. That is, the minimum interspecific distance is not higher than the maximum intraspecific distance, which contradicts the criterion of species identification with sequences distance^17^. In this context, multiple species identification methods should be incorporated to barcoding species.

Recently, it was proposed that incorporation of multiple lines of methodologies were more useful for barcoding species^26^,^27^,^28^,^41^. In these previous studies, one or two gene loci were conducted through incorporation of several barcoding approaches. In this study, four gene loci and their combined data were employed to give more evidence on the species identification. It was indicated that: (1) the GMYC model and PTP analysis generated more genetic groups; (2) the ABGD approach always recovered various genetic groups among the single marker and combined marker data; (3) as expected, all the four single data and three combined data produced consistent groups in the character-based analysis. Similar to previous studies^22^,^26^,^42^,^43^, GMYC typically generates more OTUs (operational taxonomic units) than other methods for *rbcL*, ITS, *tufA* and the combined data, and errors in the ultrametric gene tree that underpins the analysis will influence final results. The GMYC results, produced by 16S gene which may not be suitable for barcoding plantae due to the very low rates of substitution^37^, however, were relatively congruent with the resolution produced by phylogenetic analysis and other barcoding methods. It could be inferred that the GYMC method which has a strong theoretical basis may be more suitable for analyzing gene sequences evolving slowly. The PTP analysis generated various resolution among the single genes and the combined data. As the GMYC resolved, the PTP method also generated more taxonomic groups and oversplitted some species in *rbcL* and the combined data. On the other hand, the PTP analysis of *tufA*, ITS and 16S could not discriminate most species. The ABGD generated diverse outcomes among the four gene loci and the combined data, which not only over-split some certain lineages but also clustered together some lineages. As a whole, some species could be separated by ABGD analysis in all markers, but some groups generated by ABGD were not consistent with analysis of other barcoding methods. For all of *rbcL*, ITS, 16S and *tufA* genes, P ID (Liberal) could recognize most taxonomic species inferred from the phylogeetic trees, including the potentially cryptic lineages. However, only for the *tufA* and combined data sequences, all the diverged lineages were recovered as monophyletic clades. In *rbcL*, ITS and 16S genes, some diverged lineages were not recovered as monophyletic clades, e.g. the potentially cryptic lineage *S. deserticola* I. P ID (Liberal) species designation probabilities were found to be moderate significant (P > 50%) for all redefined species except some species in 16S. In sum, none of P ID, ABGD, GMYC and PTP approaches produced completely congruent clades among the single and the combined genes, but to some extent, they still could provide useful information for identification of some species.

Based on the integrated results of initial morphological characters, traditional barcoding (NJ, Bayesian and Maximum Likelihood analysis), GMYC, ABGD, P ID and PTP analysis, the putative species were confirmed by character-based method. As expected, the character-based analysis generated relatively congruent results of species discrimination in single marker of *rbcL*, ITS, 16S, *tufA* and the three combined data. All species revealed by character-based analysis possessed more than three unique character attributes. Most importantly, most taxonomic groups recovered by the character analysis were consistent with the morphological identification. Some species that were difficultly identified by morphological characters could be confirmed in character-based analysis, especially for the potential cryptic lineages. All taxonomic groups analyzed by *rbcL*, ITS, 16S, *tufA*, *rbcL* + ITS + 16S + *tufa*, *rbcL* + *tufA* and ITS + 16S, including the potential cryptic species, possessed unique simple identifying character states in character-based barcoding. Some species that could not be identified consistently with traditional barcoding, GMYC, ABGD, P ID or PTP methods could be detected by character-based method, e.g. *S. deserticola* I in *rbcL* and ITS analysis, *S. dimorphus* and *S. bajacalifornicus* in *tufA* analysis, *S. obliquus* IV in ITS analysis and *S. obliquus* I,II,III in 16S analysis. The three combined data particularly distinguished the species clearly with more attribute characters by the character-based barcoding. Generally, the groups recovered by P ID were congruent with the character analysis. Therefore, this study proved that the character-based method showed more advantages and was the most effective barcoding approach for identifying microalgae. It may be an optimal option to first combine multiple barcoding approaches to test primary species and then confirm the taxonomic assignments by the character-based method.

## Conclusions

Here we report the comprehensive molecular taxonomic identification of *Scenedesmus* to give a test that how DNA barcoding can be more effective in microalgae diversity revelation based on *rbcL*, ITS, 16S, *tufA*, *rbcL* + ITS + 16S + *tufA*, *rbcL* + *tufA* and ITS + 16S, with GMYC, P ID, PTP, ABGD and character-based barcoding approaches. First of all, the comprehensive results gave new insights into the taxonomic assignment of *Scenedesmus*, including the discrimination of most *Scenedesmus* species and the revelation of potential cryptic species. Five species, *S. deserticola*, *S. obliquus*, *S. quadricauda*, *S. spinosus* and *S. acuminatus* which were divided into several separate clades in multiple barcoding analysis of the single and combined data, could be as potential cryptic species. The three combined data showed a much higher proportion of resolution success than the single data. The traditional barcoding, GMYC, P ID, PTP and ABGD analysis of single genes generated various resolution. The character-based barcoding was proved to be the most effective approach for distinguishing species, which produced consistent species discrimination in all single and combined data and could distinguish the species clearly. After the initial morphological identification, it may be an optimal option to first combine multiple barcoding approaches to test primary microalgae species and then confirm the taxonomic assignments by the character-based method based on the single and combined data of multiple genes.

## Methods

### Algal sampling, culturing and morphological identification

The *Scenedesmus* green microalgae strains studied were collected from different environmental regions of China, e.g. the freshwaters and terrestrial areas (Fig. 1). The strains were isolated following Andersen (2005). The nonaxenic strains were grown in 250 mL flask containing 200 mL Bourelly medium at an irradiance of 40 umol m^−2^ s^−1^ with 14:10 h light: dark cycle at 20 °C. A detailed list of taxa studied, including the species name and distribution, was shown in Supplementary Table 1.

Firstly the *Scenedesmus* samples collected were identified by available morphological characters using microscope. Strains that had similar morphological characters and were difficult identified were just labeled as potential cryptic spices which would be further analyzed by the barcoding. Finally, 11 species were identified as known and 2 species were identified as unknown.

### Molecular protocols and alignment

DNA extractions were performed using the Qiagen DNEasy Plant Extraction kit (Qiagen Inc., Valencia, CA, USA). The *rbcL*, *tufA*, ITS and 16S barcode regions were amplified using either universal primers from previous studies^44^,^45^,^46^,^47^ or primers designed in the course of this study (Supplementary Table 16). PCR reactions were carried out in a total volume of 25 μL, using 2 × Taqman PCR MasterMix. PCR conditions for all primer sets were as follows: 95 °C for 3 min, primer-specific annealing temperatures for 45 s, 72 °C for 1 min; 35 cycles of 95 °C for 30 s, primer-specific annealing temperatures for 45s, 72 °C for 1 m, with a final extension of 72 °C for 1 min. The PCR products that provided a single band of sufficient intensity after running a 1.5% agarose gel were sent to the Beijing Genomics Institute (BGI) for bidirectional sequencing.

All sequences were manually edited using the program Sequencher 4.5 (Genecodes Corporation, Ann Arbor, MI). Sequences were aligned with MAFFT 6.717^48^, followed by minor adjustment if needed. Kimura 2-Parameter corrected distances^10^ between specimens were calculated with MEGA 5^49^. After edition, the *rbcL*, *tufA*, ITS and 16S sequences were combined as *rbcL* + *tufA* + ITS + 16S, the *rbcL* and *tufA* sequences were combined as *rbcL* + *tufa*, and the ITS and 16S sequences were combined as ITS + 16S.

### Data analysis

The *rbcL*, *tufA*, ITS and 16S sequences were analyzed respectively. Then the combination of *rbcL* + *tufA* + ITS + 16S, *rbcL* + *tufA* and ITS + 16S was analyzed.

The NJ analyses were conducted using Kimura 2-parameter (K2P) distance model as recommended by Hebert *et al.*^10^ in MEGA 5.0^49^ with bootstrap values (1000 replications. Bayesian trees of *rbcL*, *tufA*, ITS and 16S were generated in MrBayes v.3.1.2^50^. Nucleotide substitution models of each gene for Bayesian analyses were selected separately using the Akaike Information Criterion (AIC) as implemented in the jModeltest v.0.1.1^51^. The most appropriate models for Bayesian analyses were GTR + G for *rbcL*, GTR + G for ITS, TVMef + I + G for 16S, GTR + G for *tufa*, GTR + G for *rbcL* + *tufA* + ITS + 16S, HKY for *rbcL* + *tufa* and GTR for ITS + 16S. Four chains were run twice in parallel for 10^5^ generations with a sample frequency of 1/1,000. Maximum Likelihood trees were inferred from *rbcL*, *tufA*, ITS and 16S datasets by employing PhyML 3.0^52^. To assess the distance variation, the analyses of intra- and interspecific divergences were conducted among the final taxa assignments based on all P ID, ABGD, GMYC, PTP and CAOS analyses.

To assess species boundary hypotheses across the Bayesian gene tree, the Species Delimitation plugin^21^ within Geneious Pro v5.5.4 (Biomatters; http://www.geneious.com) was investigated. Geneious is a bioinformatics desktop software package produced by Biomatters Ltd ( http://www.biomatters.com). P ID(Liberal) in Geneious, represents the probability of making a correct identification of an unknown specimen by measuring the genetic variation found within its putative species group^27^. Maximum Likelihood trees were inferred from *rbcL*, *tufA*, ITS, 16S, *rbcL* + *tufA* + ITS + 16S, *rbcL* + *tufA* and ITS + 16S datasets by employing PhyML 3.0^52^.

A linearised Bayesian phylogenetic tree was firstly calculated in BEAST^53^ employing a Yule pure birth model tree prior. Settings in BEAUTi v. 1.7.1 were: substitution models for each gene, empirical base frequencies, four gamma categories, all codon positions partitioned with unlinked base frequencies and substitution rates. An uncorrelated relaxed lognormal clock model was used with rate estimated from the data and ucldmean parameter with uniform prior to value 0 as a lower and 10 as an upper boundary. All other settings were left as defaults. The length of MCMC chain was 40 000 000 sampling every 4000. All BEAST runs were executed in Bioportal^54^, and the ESS values and trace files of runs were evaluated in Tracer v1.5.0. Two independent runs were merged using Log-Combiner v1.7.1 with 20% burn-in. Maximum clade credibility trees with a 0.5 posterior probability limit, and node heights of target tree were constructed in TreeAnnotator v1.7.1. Single-threshold GMYC analyses was conducted in R^55^ using the APE^56^ and SPLITS^57^ packages.

The Automated Barcode Gap Discovery (ABGD) method (available at http://wwwabi.snv.jussieu.fr/public/abgd/) was used to statistically detect barcode gaps and identify distinct clusters of DNA sequences. The prior for the maximum value of intraspecific divergence was set between 0.001 and 0.1.

For Poisson tree process model (PTP), since the ultrametric trees are not required as input this coalescent-based method is very fast. This method is implemented in a web server ( http://species.h-its.org/).

The character-based identification was conducted in characteristic attribute organization system (CAOS) and CAOS-Analyzer ( http://bol.uvm.edu/caos-workbench/)^34^,^58^. The CAOS algorithm extracts characteristic attributes (CAs) for each clade at branching node within a guide tree that is first produced from a given dataset^33^. The incorporated NEXUS datasets of *rbcL*, *tufA*, ITS,16S, *rbcL* + *tufA* + ITS + 16S, *rbcL* + *tufA* and ITS + 16S NJ trees and their DNA data matrix were produced in MacClade v4.06^59^, and were carried out in CAOS system. The characteristic attributes at the nucleotide positions where the most variable sites can distinguish all the taxa were listed.

## Additional Information

**How to cite this article**: Zou, S. *et al.* How DNA barcoding can be more effective in microalgae identification: a case of cryptic diversity revelation in *Scenedesmus* (Chlorophyceae). *Sci. Rep.* **6**, 36822; doi: 10.1038/srep36822 (2016).

**Publisher’s note:** Springer Nature remains neutral with regard to jurisdictional claims in published maps and institutional affiliations.

## Supplementary Material

Supplementary Information

## Figures and Tables

**Table 1:**
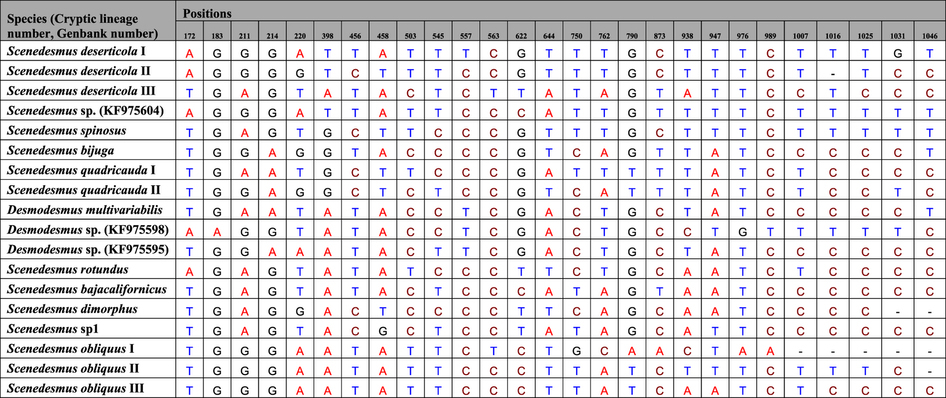
Combinations of diagnostic nucleotides for each of the 18 *Scenedesmus* taxa recovered in Fig. 2 by CAOS.

Nucleotide numbers refer to 27 selected positions on the *rbcL* sequences.

**Table 2:**
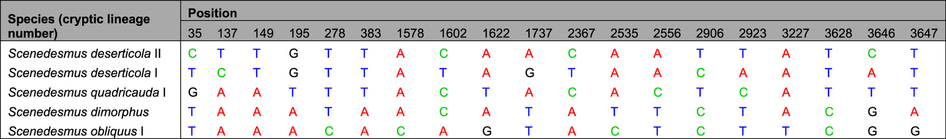
Combinations of diagnostic nucleotides for each of the 5 *Scenedesmus* taxa recovered in Fig. 3 by CAOS analysis.

Nucleotide numbers refer to 19 selected positions on the *rbcL* + ITS + 16S + *tufA* sequences.

**Table 3:**
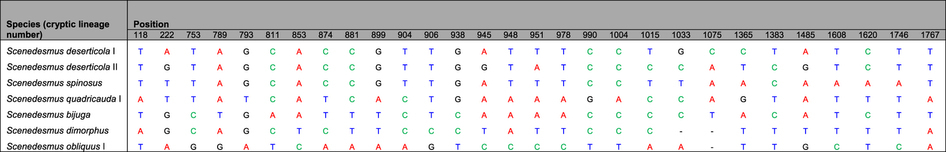
Combinations of diagnostic nucleotides for each of the 7 *Scenedesmus* taxa recovered in Fig. 4 by CAOS analysis.

Nucleotide numbers refer to 29 selected positions on the *rbcL* + *tufA* sequences.

**Table 4:**
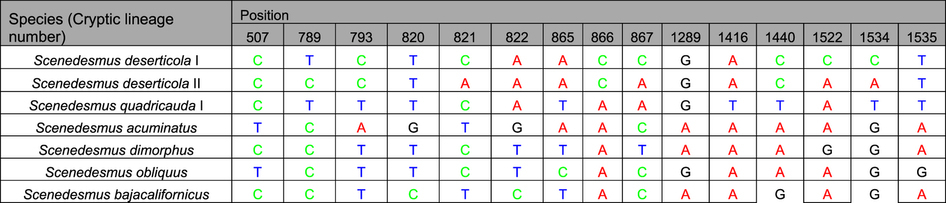
Combinations of diagnostic nucleotides for each of the 7 *Scenedesmus* taxa recovered in Fig. 5 by CAOS analysis.

Nucleotide numbers refer to 15 selected positions on the ITS +16S sequences.
