# Supplementary material for: How DNA barcoding can be more effective in microalgae identification: a case of cryptic diversity revelation in Scenedesmus (Chlorophyceae)
Source: Sci Rep. 2016 Nov 9;6:36822. doi: 10.1038/srep36822 (PMC5101840; doi:10.1038/srep36822)
Supplement: Supplementary Information [file srep36822-s1.pdf]

How DNA barcoding can be more effective in microalgae identification: a case of cryptic diversity revelation in *Scenedesmus* (Chlorophyceae)

Shanmei Zou, Cong Fei, Chun Wang, Zhan Gao, Yachao Bao, Meilin He, and Changhai Wang*

College of Resources and Environmental Science, Nanjing Agricultural University, Nanjing 210095, PR China

* Corresponding author. E-mail address: chwang@njau.edu.cn (Changhai Wang)

Supplementary Table 1. List of specimens with the classification, collection locality, and voucher numbers. GenBank accession numbers are also given, some of which in bold were previously published.

			**GenBank accession number**			
**Strain designation**	**Collection locality**	**Species**	***rbcL***	**ITS**	**16S**	***tufA***
		Scenedesmus				
SM20-1	Lake Hulunbeier, Neimeng, China	*S. deserticola*	KT777944	KT778079	KT778036	KT778003
SM20-3	Lake Hulunbeier, Neimeng, China	*S. deserticola*	KT777945	KT778078	KT778037	
SM20-4	Soil from Shihezi, Xinjiang, China	*S. deserticola*	KT777950		KT778038	
SM20_2	Lake Hulunbeier, Neimeng, China	*S. deserticola*			KT778039	KT778004
SM2_4	Lake Hulunbeier, Neimeng, China	*S. deserticola*	KT777960	KT778060		
SM1_3	Lake Hulunbeier, Neimeng, China	*S. deserticola*	KT777959	KT778076	KT778040	KT777998
SM16_2	Lianyungang, China	*S. deserticola*	KT777955	KT778067		
SM3_3	Lake Hulunbeier, Neimeng, China	*S. deserticola*	KT777946	KT778068	KT778045	KT778001
SM5_3	Lake Hulunbeier, Neimeng, China	*S. deserticola*		KT778077		
SM3_2	Soil from Shihezi, Xinjiang, China	*S. deserticola*		KT778063	KT778049	KT778000
SM3_4	Lake Taihu, Jiangsu, China	*S. deserticola*	KT777949	KT778073	KT778054	
SM4_3	Lake Taihu, Jiangsu, China	*S. deserticola*	KT777965	KT778059	KT778046	
SM5_4	Lake Taihu, Jiangsu, China	*S. deserticola*	KT777966	KT778057	KT778057	
SM3_1	Lake Taihu, Jiangsu, China	*S. deserticola*	KT777967	KT778062	KT778053	
SM2_1	Lake Zixia, Jiangsu, China	*S. deserticola*	KT777968	KT778072	KT778050	
SM5_2	Lake Taihu, Jiangsu, China	*S. deserticola*	KT777969	KT778121	KT778056	
SM5_1	Lake Taihu, Jiangsu, China	*S. deserticola*	KT777970	KT778058	KT778055	
SM4_1	Lake Donghu, Wuhan, China	*S. deserticola*	KT777971	KT778061	KT778047	KT777999
SM1_2	Lake Donghu, Wuhan, China	*S. deserticola*	KT777972	KT778074	KT778042	KT777997
SM4_2	Lake Donghu, Wuhan, China	*S. deserticola*	KT777973	KT778069	KT778051	KT778002

SM2_3	Lake Donghu, Wuhan, China	*S. deserticola*	KT777974	KT778064	KT778048	
SM1_4	Lake Donghu, Wuhan, China	*S. deserticola*	KT777975	KT778075	KT778052	KT777996
6JQ_2	Lake Mochou, Nanjing, China	*S. deserticola*	KT777976	KT778066	KT778044	
SM6_2	Lake Mochou, Nanjing, China	*S. deserticola*	KT777963			
SM4_4	Lake Mochou, Nanjing, China	*S. deserticola*	KT777964	KT778065	KT818711	
SM2_2	Lake Mochou, Nanjing, China	*S. deserticola*	KT777985	KT778071	KT778043	
SM1_1	Lake Zixia, Jiangsu, China	*S. deserticola*	KT777986	KT778070	KT778041	KT777995
BCP-YPG		*S. deserticola*	**HQ246361**			**HQ246381.1**
BCPEM2VF30		*S. deserticola*	**HQ246358**			**HQ246380.1**
		S. deserticola				**HQ246379.1**
		S. deserticola				**HQ246378.1**
		S. deserticola				**HQ246377.1**
1283_1	Xiamen, Fujian, China	*S. spinosus*	KT777977	KT778087		KT778006
1268_1	Lake Taihu, Jiangsu, China	*S. spinosus*		KT778091		KT778007
1268_2	Lake Hulunbeier, Neimeng, China	*S. spinosus*		KT778090		KT778009
1268_3	Lake Boyang, China	*S. spinosus*		KT778092		KT778008
1268_4	Lake Taihu, China	*S. spinosus*		KT778089		KT778010
1268_5	Nanjing, China	*S. spinosus*		KT778088		
44_1	Lake Hulunbeier, Neimeng, China	*S. quadricauda*	KT777982	KT778084	KT818697	
44_2	Soil from Shihezi, Xinjiang, China	*S. quadricauda*	KT777980	KT778085		
44_4	Lake Hulunbeier, Neimeng, China	*S. quadricauda*	KT777983	KT778086	KT818699	KT778012
4_2	Lake Hulunbeier, Neimeng, China	*S. quadricauda*	KT777981			
4_3	Lake Mochou, Nanjing, China	*S. quadricauda*	KT777961			
40_3	Lake Mochou, Nanjing, China	*S. quadricauda*	KT777979		KT818698	

		S. quadricauda	**AB084332**			
76_1	Donghu, Wuhan, China	*S. bijuga*	KT777978	KT778082		KT778034
76_2	Lake Taihu, Jiangsu, China	*S. bijuga*		KT778080		KT778005
76_4	Lake Zixia, Jiangsu, China	*S. bijuga*		KT778081		
		S. rotundus	**HQ246351**			**HQ246371**
		S. rotundus	**HQ246350**			**HQ246372**
959_1	Lake Yueya, Nanjing, China	*S. dimorphus*	KT777956			KT778033
959_2	Lake Yueya, Nanjing, China	*S. dimorphus*	KT777958	KT778101	KT818705	KT778013
959_3	Lake Mochou, Nanjing, China	*S. dimorphus*	KT777984	KT778102		KT778014
959_4	Ningbo, Zhejiang, China	*S. dimorphus*	KT777957	KT778103	KT818706	KT778024
959_5						KT778027
12_1	Lake Donghu, Wuhan, China	*S. obliquus*	KT777952		KT818707	KT778016
12_2	River, Nanjing, China	*S. obliquus*	KT777954	KT818720	KT818703	KT778026
12_3	Lake Xuanwu, Nanjing, China	*S. obliquus*	KT777951	KT778109		KT778015
12_4	Lake Xuanwu, Nanjing, China	*S. obliquus*	KT777953	KT778105	KT818704	KT778018
12_5	Lake Hulunbeier, Neimeng, China	*S. obliquus*				KT778017
SM17_1	Soil from Shihezi, Xinjiang, China	*S. obliquus*	KT777962	KT778104	KT818719	
SM17_4	Ningde, China	*S. obliquus*	KT777948	KT778106	KT818718	
SM14_3	Lake Hulunbeier, Neimeng, China	*S. obliquus*		KT778117		
		S. obliquus			**EU073192.1**	
		S. obliquus		**FR865721.1**		
		S. obliquus		**FR865719.1**		

		S. obliquus		**FR865722.1**		
		S. obliquus		**FR865726.1**		
		S. obliquus		**FR865731.1**		
		S. obliquus		**FR865738.1**		
		S. obliquus		**FR865737.1**		
		S. obliquus		**KJ676128.1**		
		S. obliquus				**DQ396875.1**
		S. pectinatus		**FR865723.1**		
		S. pectinatus		**FR865730.1**		
		S. regularis		**FR865732.1**		
SM9_1	Lake Hulunbeier, Neimeng, China	*S. bajacalifornicus*		KT778119	KT818700	KT778021
SM14_1	Zhoushan, Zhejiang, China	*S. bajacalifornicus*				KT778030
SM14_4	Soil from Shihezi, Xinjiang, China	*S. bajacalifornicus*		KT778118		KT778029
SM14_2	Lake Hulunbeier, Neimeng, China	*S. bajacalifornicus*		KT778116		
SM9_5	Lake Hulunbeier, Neimeng, China	*S. bajacalifornicus*		KT778115	KT818702	KT778028
SM9_4	Lake Hulunbeier, Neimeng, China	*S. bajacalifornicus*		KT778114		KT778031
SM9_2	Lianyungang, China	*S. bajacalifornicus*		KT778113		KT778019
SM9_3	Lake Hulunbeier, Neimeng, China	*S. bajacalifornicus*			KT818701	KT778035
SM13_4	Lake Hulunbeier, Neimeng, China	*S. bajacalifornicus*		KT778112	KT818717	KT778020
SM13_3	Soil from Shihezi, Xinjiang, China	*S. bajacalifornicus*		KT778111		KT778023
SM13_1	Lake Taihu, Jiangsu, China	*S. bajacalifornicus*				KT778025
SM13_2	Lake Xuanwu, Jiangsu, China	*S. bajacalifornicus*		KT778110	KT818716	KT778032
		S. bajacalifornicus	**KF975592**		**EU073189.1**	**HQ246375.1**

		S. bajacalifornicus	**KF975602**			**HQ246374.1**
		S. bajacalifornicus	**HQ246355**			**HQ246376.1**
		S. bajacalifornicus	**HQ246354**			
		S. bajacalifornicus	**HQ246357**			
		S. bajacalifornicus	**HQ246353**			
1221_1	River, Lianyungang, China	*S. acuminatus*		KT778100	KT818709	KT778022
1221_4	River, Nanjing, China	*S. acuminatus*		KT778099	KT818708	
1271_3	Lake Mochou, Nanjing, China	*S. acuminatus*			KT818710	
SM8_2	Lake Hulunbeier, Neimeng, China	*S.* sp	KT777947	KT778097		
SM8_1	Lake Taihu, Jiangsu, China	*S.* sp		KT778095		
SM8_3	Lake Taihu, Jiangsu, China	*S.* sp		KT778096		
SM8_4	Lake Xuanwu, Jiangsu, China	*S.* sp		KT778098		
SM15_1	Lake Taihu, Jiangsu, China	*S.* sp		KT778093		
SM15_4	Lake Donghu, Wuhan, China	*S.* sp		KT778094		KT778011
		S. sp	**KF975604**			
		S. sp			**EU073191.1**	
		S. sp			**EU073193.1**	
		S. sp			**EU073194.1**	
		S. sp			**EU073195.1**	
		S. sp			**AJ548893.1**	
		S. sp			**AJ548894.1**	
		S. sp			**KC994749.1**	
		S. sp			**GQ920625.1**	
		S. sp			**AF394206.1**	

		S. sp			**KC994774.1**	
		S. sp			**KC994812.1**	
		S. sp			**KC994840.1**	
		S. sp			**KC994867.1**	
AKS-19		*S.* sp	**KF975603.1**			
					**AJ548895.1**	
Outgroup						
		Chlorella				
275_4	Lake Xuanwu, Nanjing, China	*C. sorokiniana*	KT777992	KT778083	KT818713	KR154271
275_1	Qingdao, China	*C. sorokiniana*	KT777993	KT778120	KT818712	KR154269
275_2	Xiamen, Fujian, China	*C. sorokiniana*	KT777994	KT778107		KR154270
5_4	Lake Donghu, Wuhan, China	*C. ellipsoidea*	KT777991	KT778122		
5_3	Soil from Shihezi, Xinjiang, China	*C. ellipsoidea*	KT777990		KT818715	
5_2	Lake Mochou, Jiangsu, China	*C. ellipsoidea*	KT777989	KM514842	KT818714	
1_1	River, Wuhan, China	*C. ellipsoidea*	KT777987			
2_2	Lake Yueya, Nanjing, China	*C. ellipsoidea*	KT777988			
CCAP 211/11M		*C. emersonii*		**FR865657**		
CCAP 211/15		*C. emersonii*		**FR865661**		
IAM C-101		*C.pyrenoidosa*			**AJ242752.1**	
		C.pyrenoidosa			**JN887917.1**	
		Desmodesmus				
Mary 8/18 T-1w		*D.multivariabilis*	**GU192431**			

AKS-11	*D.* sp	**KF975598**
AKS-7	*D.* sp	**KF975595.1**

Supplementary Table S2. The mean interspecific divergences of *rbcL* sequences for *Scenedesmus* taxa (lower left: nucleotide divergences, upper right: standard error). The taxa name corresponds to the assignments in Figure 2.

Taxa		1	2	3	4	5	6	7	8	9	10	11	12	13	14	15	16	17	18
Clade_10	1		0.010	0.011	0.009	0.009	0.010	0.009	0.010	0.011	0.011	0.013	0.013	0.013	0.013	0.013	0.012	0.012	0.019
Clade_5	2	0.082		0.005	0.008	0.010	0.010	0.009	0.009	0.010	0.010	0.012	0.011	0.012	0.012	0.012	0.012	0.011	0.019
Clade_4	3	0.086	0.023		0.009	0.010	0.010	0.009	0.008	0.009	0.009	0.012	0.012	0.011	0.011	0.012	0.011	0.010	0.019
Clade_6	4	0.072	0.060	0.068		0.010	0.011	0.010	0.011	0.011	0.011	0.013	0.012	0.012	0.012	0.013	0.011	0.011	0.019
Clade_8	5	0.065	0.073	0.077	0.073		0.002	0.004	0.008	0.010	0.010	0.011	0.011	0.012	0.012	0.012	0.012	0.011	0.019
Clade_9	6	0.067	0.078	0.082	0.074	0.004		0.005	0.008	0.010	0.010	0.011	0.011	0.012	0.012	0.012	0.012	0.011	0.019
Clade_7	7	0.062	0.069	0.071	0.068	0.016	0.017		0.007	0.009	0.009	0.012	0.010	0.012	0.011	0.011	0.011	0.010	0.018
Clade_3	8	0.083	0.076	0.056	0.093	0.065	0.063	0.051		0.006	0.005	0.012	0.011	0.011	0.012	0.012	0.011	0.010	0.018
Clade_2	9	0.093	0.082	0.067	0.095	0.075	0.074	0.058	0.037		0.007	0.013	0.009	0.011	0.010	0.010	0.010	0.010	0.017
Clade_1	10	0.088	0.081	0.060	0.089	0.077	0.076	0.064	0.032	0.036		0.013	0.011	0.011	0.011	0.011	0.011	0.010	0.018
Clade_11	11	0.108	0.094	0.093	0.115	0.093	0.093	0.096	0.106	0.104	0.106		0.010	0.010	0.009	0.010	0.012	0.011	0.019
Clade_18	12	0.105	0.089	0.089	0.107	0.089	0.092	0.079	0.089	0.071	0.089	0.067		0.008	0.006	0.006	0.009	0.009	0.016
Clade_12	13	0.101	0.098	0.087	0.108	0.096	0.099	0.095	0.092	0.090	0.091	0.068	0.046		0.007	0.007	0.009	0.009	0.017
Clade_17	14	0.105	0.095	0.087	0.105	0.095	0.098	0.086	0.094	0.077	0.086	0.067	0.030	0.034		0.004	0.009	0.009	0.017
Clade_16	15	0.104	0.098	0.093	0.108	0.096	0.099	0.087	0.092	0.078	0.092	0.068	0.028	0.037	0.012		0.009	0.009	0.016
Clade_15	16	0.098	0.094	0.087	0.095	0.099	0.099	0.087	0.088	0.081	0.089	0.090	0.055	0.058	0.058	0.057		0.007	0.015
Clade_14	17	0.102	0.089	0.078	0.090	0.090	0.092	0.078	0.076	0.069	0.070	0.083	0.055	0.059	0.055	0.054	0.035		0.014
Clade_13	18	0.228	0.226	0.216	0.223	0.228	0.230	0.214	0.213	0.203	0.216	0.205	0.168	0.178	0.173	0.166	0.156	0.136	

Supplementary Table S3. The mean interspecific divergences of ITS sequences for *Scenedesmus* taxa (lower left: nucleotide divergences, upper right: standard error). The taxa name corresponds to the assignments in Supplementary Figure 6.

Taxa		1	2	3	4	5	6	7	8	9	10	11	12	13
Clade_1	1		0.003	0.007	0.009	0.009	0.016	0.015	0.015	0.015	0.015	0.014	0.015	0.015
Clade_2	2	0.011		0.008	0.009	0.009	0.017	0.015	0.015	0.015	0.015	0.015	0.015	0.015
Clade_3	3	0.046	0.053		0.008	0.008	0.017	0.015	0.016	0.015	0.016	0.015	0.015	0.015
Clade_4	4	0.073	0.083	0.070		0.009	0.017	0.016	0.016	0.015	0.016	0.015	0.016	0.015
Clade_5	5	0.066	0.071	0.058	0.084		0.017	0.016	0.016	0.016	0.017	0.016	0.016	0.016
Clade_8	6	0.189	0.192	0.208	0.217	0.201		0.010	0.012	0.011	0.010	0.010	0.010	0.009
Clade_6	7	0.162	0.165	0.165	0.193	0.179	0.093		0.011	0.009	0.009	0.009	0.008	0.008
Clade_12	8	0.171	0.171	0.179	0.199	0.190	0.118	0.096		0.009	0.008	0.007	0.007	0.008
Clade_13	9	0.166	0.169	0.170	0.189	0.182	0.099	0.070	0.059		0.007	0.006	0.006	0.006
Clade_11	10	0.159	0.163	0.169	0.184	0.179	0.088	0.068	0.049	0.038		0.004	0.005	0.004
Clade_10	11	0.156	0.160	0.161	0.185	0.174	0.089	0.067	0.045	0.032	0.016		0.004	0.003
Clade_14	12	0.157	0.160	0.161	0.188	0.175	0.087	0.064	0.044	0.031	0.018	0.013		0.003
Clade_9	13	0.160	0.164	0.166	0.186	0.180	0.083	0.064	0.050	0.033	0.019	0.011	0.012	

Supplementary Table S4. The mean interspecific divergences of 16S sequences for *Scenedesmus* taxa (lower left: nucleotide divergences, upper right: standard error). The taxa name corresponds to the assignments in Supplementary Figure 9.

Taxa		1	2	3	4	5	6	7	8	9	10	11	12	13	14	15
Clade_1	1		0.030	0.032	0.031	0.031	0.037	0.038	0.040	0.039	0.039	0.040	0.041	0.039	0.039	0.039
Clade_2	2	0.237		0.011	0.011	0.010	0.018	0.017	0.018	0.018	0.018	0.021	0.020	0.019	0.018	0.018
Calde_3	3	0.269	0.048		0.010	0.012	0.018	0.017	0.020	0.020	0.019	0.022	0.022	0.020	0.019	0.019
Clade_4	4	0.258	0.046	0.036		0.014	0.017	0.017	0.018	0.018	0.018	0.021	0.021	0.020	0.018	0.018
Clade_5	5	0.262	0.037	0.051	0.064		0.018	0.016	0.016	0.017	0.016	0.019	0.019	0.018	0.016	0.017
Clade_6	6	0.318	0.106	0.108	0.103	0.103		0.016	0.016	0.017	0.016	0.019	0.019	0.019	0.016	0.016
Clade_14	7	0.325	0.095	0.101	0.102	0.083	0.086		0.007	0.009	0.008	0.011	0.011	0.009	0.008	0.008
Calde_13	8	0.344	0.102	0.117	0.111	0.080	0.083	0.020		0.007	0.006	0.010	0.008	0.011	0.006	0.006
Clade_12	9	0.338	0.100	0.120	0.115	0.088	0.097	0.034	0.022		0.007	0.011	0.011	0.012	0.007	0.007
Clade_11	10	0.338	0.102	0.107	0.102	0.086	0.080	0.023	0.014	0.017		0.008	0.010	0.011	0.000	0.001
Calde_10	11	0.351	0.133	0.138	0.133	0.116	0.110	0.050	0.042	0.045	0.027		0.008	0.009	0.008	0.008
Clade_9	12	0.350	0.128	0.143	0.138	0.105	0.108	0.043	0.023	0.045	0.037	0.031		0.010	0.010	0.010
Clade_15	13	0.334	0.118	0.123	0.125	0.105	0.109	0.026	0.040	0.055	0.043	0.039	0.036		0.011	0.011
Clade_8	14	0.338	0.102	0.107	0.102	0.086	0.080	0.023	0.014	0.017	0.000	0.027	0.037	0.043		0.001
Clade_7	15	0.339	0.103	0.108	0.103	0.087	0.081	0.024	0.016	0.019	0.002	0.029	0.039	0.045	0.002	

Supplementary Table S5. The mean interspecific divergences of *tufA* sequences for *Scenedesmus* taxa (lower left: nucleotide divergences, upper right: standard error). The taxa name corresponds to the assignments in Supplementary Figure 3.

Taxa		1	2	3	4	5	6	7	8	9	10	11	12	13	14
Clade_1	1		0.009	0.015	0.042	0.035	0.036	0.023	0.023	0.015	0.023	0.024	0.023	0.023	0.026
Clade_2	2	0.024		0.014	0.042	0.033	0.034	0.022	0.022	0.017	0.021	0.022	0.022	0.022	0.025
Clade_3	3	0.068	0.057		0.041	0.033	0.034	0.019	0.021	0.016	0.019	0.020	0.019	0.019	0.022
Clade_4	4	0.363	0.368	0.357		0.039	0.037	0.043	0.040	0.041	0.041	0.042	0.042	0.043	0.043
Clade_5	5	0.270	0.255	0.250	0.325		0.021	0.038	0.038	0.035	0.033	0.034	0.034	0.034	0.037
Clade_6	6	0.300	0.278	0.291	0.320	0.139		0.037	0.035	0.034	0.033	0.032	0.033	0.033	0.034
Clade_7	7	0.147	0.139	0.111	0.380	0.295	0.312		0.017	0.021	0.020	0.020	0.019	0.019	0.020
Clade_8	8	0.139	0.127	0.131	0.368	0.300	0.295	0.083		0.021	0.019	0.019	0.021	0.020	0.021
Clade_9	9	0.064	0.083	0.076	0.374	0.280	0.296	0.119	0.127		0.019	0.019	0.019	0.019	0.021
Clade_10	10	0.139	0.119	0.107	0.352	0.259	0.279	0.110	0.107	0.095		0.008	0.010	0.009	0.012
Clade_11	11	0.148	0.127	0.115	0.364	0.265	0.271	0.115	0.107	0.103	0.021		0.010	0.009	0.012
Clade_13	12	0.135	0.131	0.103	0.357	0.265	0.287	0.107	0.123	0.099	0.031	0.032		0.007	0.012
Clade_14	13	0.147	0.127	0.107	0.371	0.269	0.282	0.108	0.118	0.109	0.030	0.029	0.019		0.010
Clade_12	14	0.168	0.147	0.127	0.368	0.290	0.290	0.111	0.119	0.115	0.043	0.039	0.042	0.039	

Supplementary Table S6. Species Delimitation Results of PID for *rbcL*. The species number corresponds to the P ID clades in Fig. 2.

Species	Closest Species	Monophyletic?	Intra Dist	Inter Dist - Closest	Intra/Inter	P ID(Strict)	P ID(Liberal)	Av(MRCA-ti	P(Randomly Distinct)	Clade Support	Rosenberg's P(AB)
1	2	yes	2.667	3.667	0.73	0.30 (0.12, 0.49)	0.60 (0.45, 0.74)	1.6667	0.09	NA	0.17
2	3	yes	0.00E+00	3.304	0.00E+00	0.00E+00 (0.00E+00, 0.00E+00)	0.96 (0.83, 1.0)	0.00E+00	NA	NA	0.17
3	2	no	2.569	3.304	0.78	0.73 (0.68, 0.79)	0.92 (0.89, 0.95)	1.3043	NA	NA	NA
4	5	yes	0.00E+00	4	0.00E+00	0.00E+00 (0.00E+00, 0.00E+00)	0.96 (0.83, 1.0)	0.00E+00	NA	NA	2.65E-03
5	4	yes	2	4	0.5	0.60 (0.47, 0.72)	0.87 (0.76, 0.97)	1	1	NA	4.50E-08
6	4	yes	0.00E+00	4	0.00E+00	0.00E+00 (0.00E+00, 0.00E+00)	0.96 (0.83, 1.0)	0.00E+00	NA	NA	1.68E-03
7	8	yes	0.00E+00	3	0.00E+00	0.00E+00 (0.00E+00, 0.00E+00)	0.96 (0.83, 1.0)	0.00E+00	NA	NA	0.33
8	9	yes	0.00E+00	2	0.00E+00	0.00E+00 (0.00E+00, 0.00E+00)	0.96 (0.83, 1.0)	0.00E+00	NA	NA	1
9	8	yes	0.00E+00	2	0.00E+00	0.00E+00 (0.00E+00, 0.00E+00)	0.96 (0.83, 1.0)	0.00E+00	NA	NA	1
10	6	yes	0.00E+00	4	0.00E+00	0.00E+00 (0.00E+00, 0.00E+00)	0.96 (0.83, 1.0)	0.00E+00	NA	NA	1.35E-03
11	10	yes	2	5	0.4	0.39 (0.23, 0.54)	0.74 (0.58, 0.89)	1	1	NA	4.10E-04
12	11	yes	2	5	0.4	0.60 (0.45, 0.74)	0.86 (0.75, 0.97)	1	NA	2.10E-05	
13	14	yes	2.5	3.75	0.67	0.42 (0.27, 0.56)	0.73 (0.62, 0.84)	1.75	NA	0.1	
14	13	yes	0.00E+00	3.75	0.00E+00	0.00E+00 (0.00E+00, 0.00E+00)	0.96 (0.83, 1.0)	0.00E+00	NA	NA	0.1
15	14	yes	2	4	0.5	0.34 (0.18, 0.49)	0.67 (0.52, 0.83)	1	1	NA	0.02
16	17	yes	2	3	0.67	0.25 (0.09, 0.41)	0.57 (0.41, 0.73)	1	NA	0.33	
17	16	yes	0.00E+00	3	0.00E+00	0.00E+00 (0.00E+00, 0.00E+00)	0.96 (0.83, 1.0)	0.00E+00	NA	NA	0.33
18	17	yes	2	4	0.5	0.60 (0.47, 0.72)	0.87 (0.76, 0.97)	1	1	NA	2.98E-03

Supplementary Table S7. Species Delimitation Results of PID for ITS. The species number corresponds to the P ID clades in Supplementary Fig. 6.

Species	Closest Species	Monophyletic?	Intra Dist	Inter Dist - Closest	Intra/Inter	P ID(Strict)	P ID(Liberal)	Av(MRCA-tips)	P(Randomly Distinct)	Clade Support	Rosenberg's P(AB)
1	2	no	2	3	0.67	0.79 (0.73, 0.84)	0.94 (0.92, 0.97)	1	NA	NA	NA
2	1	yes	2	3	0.67	0.25 (0.09, 0.41)	0.57 (0.41, 0.73)	1	NA	3.10E-04	
3	1	yes	2	5	0.4	0.52 (0.35, 0.70)	0.78 (0.63, 0.93)	1	NA	8.20E-04	
4	5	yes	2	4	0.5	0.46 (0.28, 0.64)	0.72 (0.58, 0.87)	1	NA	2.98E-03	
5	4	yes	2	4	0.5	0.60 (0.47, 0.72)	0.87 (0.76, 0.97)	1	NA	2.98E-03	
6	7	yes	2	3	0.67	0.25 (0.09, 0.41)	0.57 (0.41, 0.73)	1	NA	0.33	
7	6	yes	0.00E+00	3	0.00E+00	0.00E+00 (0.00E+00, 0.00E+00)	0.96 (0.83, 1.0)	0.00E+00	NA	NA	0.33
8	7	yes	2	4	0.5	0.34 (0.18, 0.49)	0.67 (0.52, 0.83)	1	NA	0.05	
9	10	yes	3.278	5.778	0.57	0.70 (0.62, 0.79)	0.91 (0.85, 0.96)	1.7778	NA	1.60E-09	
10	11	yes	2	5	0.4	0.67 (0.54, 0.79)	0.90 (0.80, 1.0)	1	NA	2.70E-06	
11	10	yes	2	5	0.4	0.52 (0.35, 0.70)	0.78 (0.63, 0.93)	1	NA	1.40E-04	
12	13	yes	2	4	0.5	0.53 (0.38, 0.68)	0.82 (0.71, 0.93)	1	NA	0.03	
13	12	yes	2	4	0.5	0.34 (0.18, 0.49)	0.67 (0.52, 0.83)	1	NA	0.03	
14	11	yes	2	5	0.4	0.78 (0.69, 0.86)	0.93 (0.88, 0.98)	1	0.97	NA	2.80E-05

Supplementary Table S8. Species Delimitation Results of PID for *tufA*. The species number corresponds to the P ID clades in Supplementary Fig. 3.

Species	Closest Species	Monophyletic?	Intra Dist	Inter Dist - Closest	Intra/Inter	P ID(Strict)	P ID(Liberal)	Av(MRCA-tips)	P(Randomly Distinct)	Clade Support	Rosenberg's P(AB)
1	2	yes	4.75	7.375	0.64	0.60 (0.49, 0.70)	0.86 (0.80, 0.92)	4.375	NA	4.94E-03	
2	3	yes	2	5	0.4	0.39 (0.23, 0.54)	0.74 (0.58, 0.89)	1	NA	4.94E-03	
3	2	yes	2	5	0.4	0.39 (0.23, 0.54)	0.74 (0.58, 0.89)	1	NA	2.75E-03	
4	5	yes	0.00E+00	3	0.00E+00	0.00E+00 (0.00E+00, 0.00E+00)	0.96 (0.83, 1.0)	0.00E+00	NA	NA	0.1
5	4	yes	0.00E+00	3	0.00E+00	0.00E+00 (0.00E+00, 0.00E+00)	0.96 (0.83, 1.0)	0.00E+00	NA	NA	0.17
6	5	yes	2.667	3.667	0.73	0.30 (0.12, 0.49)	0.60 (0.45, 0.74)	1.6667	NA	0.17	
7	8	yes	0.00E+00	3	0.00E+00	0.00E+00 (0.00E+00, 0.00E+00)	0.96 (0.83, 1.0)	0.00E+00	NA	NA	0.33
8	7	yes	2	3	0.67	0.25 (0.09, 0.41)	0.57 (0.41, 0.73)	1	NA	0.33	
9	7	yes	2	5	0.4	0.39 (0.23, 0.54)	0.74 (0.58, 0.89)	1	0.64	NA	4.10E-04
10	9	yes	3.167	7.25	0.44	0.57 (0.43, 0.72)	0.85 (0.74, 0.96)	2.25	0.97	NA	0.1
11	12	yes	4	6.333	0.63	0.51 (0.38, 0.64)	0.81 (0.71, 0.91)	3.3333	0.94	NA	5.60E-08
12	13	yes	0.00E+00	5.8	0.00E+00	0.00E+00 (0.00E+00, 0.00E+00)	0.96 (0.83, 1.0)	0.00E+00	NA	NA	1.78E-03
13	12	yes	3.6	5.8	0.62	0.52 (0.39, 0.64)	0.81 (0.71, 0.91)	2.8	1	NA	1.10E-08
14	12	yes	6.514	9.133	0.71	0.71 (0.65, 0.78)	0.91 (0.87, 0.96)	6.1333	1	NA	4.30E-15

Supplementary Table S9. Species Delimitation Results of PID for 16S. The species number corresponds to the P ID clades in Supplementary Fig. 9.

Species	Closest Species	Monophyletic?	Intra Dist	Inter Dist - Closest	Intra/Inter	P ID(Strict)	P ID(Liberal)	Av(MRCA-tips)	P(Randomly Distinct)	Clade Support	Rosenberg's P(AB)
1	2	yes	2.5	3.917	0.64	0.44 (0.29, 0.58)	0.75 (0.64, 0.86)	1.75	1	NA	5.80E-08
2	1	no	2.294	3.917	0.59	0.82 (0.76, 0.87)	0.95 (0.93, 0.98)	1.1667	NA	NA	NA
3	4	yes	2.667	4.667	0.57	0.41 (0.23, 0.59)	0.68 (0.54, 0.83)	1.6667	0.87	NA	0.01
4	2	yes	2	4.167	0.48	0.54 (0.40, 0.69)	0.83 (0.72, 0.94)	1	0.54	NA	0.01
5	6	yes	2	4	0.5	0.34 (0.18, 0.49)	0.67 (0.52, 0.83)	1	0.54	NA	1.70E-04
6	7	yes	0.00E+00	3	0.00E+00	0.00E+00 (0.00E+00, 0.00E+00)	0.96 (0.83, 1.0)	0.00E+00	NA	NA	2.85E-03
7	6	no	2	3	0.67	0.58 (0.47, 0.69)	0.85 (0.79, 0.92)	1	NA	NA	NA
8	11	no	2	2	1	0.08 (0.00E+00, 0.24)	0.37 (0.21, 0.53)	1	NA	NA	NA
9	10	yes	0.00E+00	2.5	0.00E+00	0.00E+00 (0.00E+00, 0.00E+00)	0.96 (0.83, 1.0)	0.00E+00	NA	NA	1
10	9	no	3	2.5	1.2	0.00E+00 (0.00E+00, 0.14)	0.24 (0.08, 0.40)	1.5	NA	NA	NA
11	8	yes	0.00E+00	2	0.00E+00	0.00E+00 (0.00E+00, 0.00E+00)	0.96 (0.83, 1.0)	0.00E+00	NA	NA	0.01
12	8	yes	2.333	3.833	0.61	0.52 (0.40, 0.65)	0.82 (0.72, 0.92)	1.8333	NA	6.30E-06	
13	8	yes	2	4	0.5	0.46 (0.28, 0.64)	0.72 (0.58, 0.87)	1	0.97	NA	0.02
14	15	yes	0.00E+00	3	0.00E+00	0.00E+00 (0.00E+00, 0.00E+00)	0.96 (0.83, 1.0)	0.00E+00	NA	NA	0.33
15	14	yes	2	3	0.67	0.25 (0.09, 0.41)	0.57 (0.41, 0.73)	1	NA	0.33	

Supplementary Table 10. Combinations of diagnostic nucleotides for each of the 14 *Scenedesmus* taxa recovered in Supplementary Fig. 6 by CAOS. Nucleotide numbers refer to 28 selected positions on the ITS sequences.

**Species (Cryptic lineage number, Genbank number)**	**Positions**																											
	**246**	**349**	**358**	**360**	**368**	**396**	**418**	**422**	**423**	**431**	**444**	**445**	**449**	**452**	**473**	**500**	**512**	**538**	**555**	**738**	**740**	**758**	**764**	**812**	**966**	**973**	**974**	**1208**
***Scenedesmus deserticola* I**	C	A	C	C	-	-	T	C	T	C	G	A	C	T	-	G	C	T	-	C	T	G	C	C	G	T	C	T
***Scenedesmus deserticola* II**	T	A	C	C	-	-	T	T	C	C	A	A	C	T	C	G	C	T	-	C	T	G	C	A	A	T	C	C
Scenedesmus bijuga	C	A	C	C	-	-	C	C	T	T	A	A	T	G	-	T	C	C	G	T	A	G	C	-	A	-	C	C
Scenedesmus quadricauda	C	A	C	C	-	-	C	C	T	C	A	C	C	G	-	G	C	T	G	T	T	G	C	A	G	T	A	C
Scenedesmus spinosus	C	A	C	C	-	-	C	T	T	C	A	A	C	G	-	A	C	T	G	T	A	G	T	-	A	-	C	C
***Scenedesmus* sp 2**	A	A	C	T	T	T	G	C	C	T	A	T	T	C	A	G	T	T	A	-	T	G	-	C	G	G	T	C
Scenedesmus pectinatus	A	A	C	T	C	A	G	C	T	T	-	-	T	C	A	G	A	C	A	T	G	G	-	G/T	A	T	C	C
Scenedesmus regularis	A	A	C	T	C	A	G	C	T	T	-	-	T	C	A	G	C	C	A	T	G	G	-	G	A	T	C	C
***Scenedesmus* sp 1**	A	T	A	C	T	C	T	C	C	T	-	T	T	T	A	G	C	C	A	C	C	A	A	C	A	T	C	G
Scenedesmus acuminatus	A	A	T	T	T	A	T	C	T	T	A	-	T	T	A	G	A	C	A	A	G	G	T	C	A	T	A	C
Scenedesmus dimorphus	A	A	T	T	A	A	T	C	T	T	-	-	-	T	A	G	C	T	A	T	T	A	T	T	A	T	C	G
***Scenedesmus obliquus* I,II,III**	A	A	T	C	T	A	T	C	T	T	A	T	T	T	A	G	C	C	A	T	T	A	T	C	A	T	C	G
***Scenedesmus obliquus* IV**	A	A	C	T	C	A	T	C	T	T	A	T	T	T	A	G	C	C	A	T	T	A	-	C	A	C	T	T
***Scenedesmus* sp 3**	A	A	C	T	T	G	T	C	T	T	A	G	T	T	A	G	C	C	A	T	T	A	T	C	A	T	C	G

Supplementary Table S11. Combinations of diagnostic nucleotides for each of the 15 *Scenedesmus* taxa recovered by 16S in Supplementary Fig. 9 by CAOS analysis. Nucleotide numbers refer to 25 selected positions on the 16S sequences.

**Species (Cryptic lineage number and Genbank number)**	**Positions**																								
	**49**	**87**	**88**	**169**	**181**	**182**	**219**	**326**	**336**	**337**	**338**	**345**	**359**	**366**	**376**	**378**	**381**	**382**	**384**	**394**	**401**	**403**	**404**	**405**	**406**
***Scenedesmus deserticola* II**	C	C	T	A	A	T	A	A	T	T	T	T	A	T	T	A	G	C	T	C	A	A	T	C	T
***Scenedesmus deserticola* I**	C	C	T	C	C	T	A	G	G	A	A	C	G	G	C	G	A	T	C	T	C	A	T	C	A
Scenedesmus quadricauda	C	T	T	A	T	T	A	C	A	A	C	G	C	G	C	T	A	T	A	T	C	A	T	C	A
***Scenedesmus* sp (EU073191.EU073193.EU073194.EU073195.)**	C	T	T	A	A	T	G	C	A	A	C	G	G	A	C	T	A	T	C	T	C	A	T	C	A
***Scenedesmus* sp (AJ548893.AJ548894.)**	C	T	T	G	T	T	A	G	T	T	A	C	G	G	C	T	A	T	C	T	C	A	T	C	A
***Scenedesmus* sp (FJ608099.)**	C	A	C	G	T	G	A	G	T	G	T	C	G	G	C	T	T	T	C	T	C	A	T	C	A
***Scenedesmus acuminatus* II**	T	A	C	A	G	A	G	G	T	T	A	C	G	C	G	T	A	T	C	T	C	A	T	C	A
***Scenedesmus acuminatus* I**	C	A	C	A	G	A	G	G	T	T	A	C	G	C	G	T	A	-	C	T	C	T	C	T	T
Scenedesmus dimorphus	C	A	C	G	G	A	G	G	T	T	A	C	G	C	G	G	A	T	C	T	C	A	T	C	A
***Scenedesmus obliquus* I- a**	C	A	C	A	G	G	G	G	T	G	A	C	G	C	G	T	A	-	C	T	T	T	A	G	C
***Scenedesmus obliquus* I- b**	C	A	C	A	G	G	G	G	T	G	A	C	G	C	G	T	A	-	C	A	A	T	C	A	T
***Scenedesmus obliquus* II**	C	A	C	A	G	G	G	G	T	G	A	C	G	C	G	T	A	T	C	T	C	A	T	C	A
***Scenedesmus obliquus* III**	C	A	C	G	G	A	G	G	T	T	A	C	G	C	G	G	A	-	C	T	T	T	T	G	C
Scenedesmus bajacalifornicus	G	G	C	A	G	A	G	G	T	G	A	C	G	C	G	T	A	T	C	T	C	A	T	C	A
***Scenedesmus obliquus* vs sp.**	C	A	C	A	G	G	T	G	T	G	A	C	G	C	G	T	A	T	C	T	C	A	T	C	A

Supplementary Table 12. Combinations of diagnostic nucleotides for each of the 14 *Scenedesmus* taxa recovered in Supplementary Fig. 3 by CAOS analysis. Nucleotide numbers refer to 39 selected positions on the *tufA* sequences.

**Species (Cryptic lineage number and Genbank number)**	**Positions**																																					
	**137**	**173**	**195**	**197**	**198**	**200**	**212**	**215**	**216**	**227**	**251**	**254**	**258**	**263**	**272**	**287**	**290**	**317**	**323**	**329**	**335**	**347**	**350**	**353**	**368**	**371**	**375**	**389**	**393**	**402**	**404**	**408**	**423**	**429**	**430**	**434**	**452**	**472**	**473**
***Scenedesmus deserticola* I**	T	A	G	A	A	C	T	C	A	C	T	T	G	C	A	A	C	T	A	C	A	A	T	A	T	T	T	A	G	G	A	T	T	G	T	T	C	C	T
***Scenedesmus deserticola* II**	C	A	G	A	A	C	T	C	A	C	T	G	G	T	A	A	C	T	A	C	A	A	T	A	T	T	T	A	G	G	A	T	T	G	T	T	C	C	T
Scenedesmus bijuga	A	A	T	T	A	C	T	T	A	T	A	T	G	T	T	A	C	T	A	C	A	A	A	A	T	A	T	A	G	G	A	T	G	G	T	A	C	C	A
***Scenedesmus spinosus* I**	T	T	G	T	C	A	C	T	C	T	T	T	G	T	T	T	A	C	T	T	T	A	C	A	T	G	G	T	G	G	T	A	A	A	C	T	T	G	A
***Scenedesmus spinosus* II**	A	A	C	A	A	C	T	T	A	T	A	T	T	T	T	A	G	C	T	T	T	A	G	A	T	G	G	T	G	G	T	T	A	A	C	T	C	G	T
***Scenedesmus spinosus* III**	A	A	G	A	A	A	T	T	A	T	A	T	T	C	T	A	G	C	T	T	C	T	T	A	T	A	G	T	G	A	T	T	A	A	C	T	T	G	C
***Scenedesmus* sp2**	A	A	T	A	G	T	A	T	A	C	A	A	A	C	A	A	A	T	A	C	T	G	A	A	A	A	T	A	G	G	T	T	T	G	T	A	C	C	A
Scenedesmus rotundus	A	C	A	A	G	T	A	C	A	T	A	T	A	C	A	A	C	T	A	C	T	A	C	T	A	A	T	A	A	G	T	T	T	G	T	C	T	C	A
Scenedesmus quadricauda	A	A	T	A	A	T	T	C	A	T	A	T	G	C	A	A	T	T	A	T	A	A	T	A	A	A	T	A	G	G	T	C	G	G	T	T	C	C	A
Scenedesmus dimorphus	A	A	A	A	A	T	T	C	C	T	A	T	G	T	A	T	T	T	A	T	T	A	A	A	A	A	T	A	G	G	T	T	G	G	T	T	C	C	A
Scenedesmus obliquus	A	A	A	A	A	T	T	C	A	T	A	T	G	T	A	T	T	T	A	T	T	T	A	A	A	A	T	A	G	A	T	T	G	G	T	T	C	C	A
Scenedesmus deserticola III	A	A	A	A	A	T	T	C	A	T	A	T	C	T	A	T	T	T	A	T	T	T	A	A	A	A	T	A	G	A	T	T	G	G	T	T	C	C	A
Scenedesmus bajacalifornicus	A	A	A	A	A	T	T	C	A	T	A	T	G	T	A	T	T	T	A	T	T	T	A	A	A	A	T	A	G	A	T	T	T	G	T	T	C	C	A
Scenedesmus acuminatus	G	G	A	A	A	T	T	C	A	T	A	T	G	T	A	T	T	T	A	T	T	G	A	A	A	A	T	A	G	A	T	T	G	G	T	T	T	C	A

Supplementary Table 13. Species Delimitation Results of PID for *rbcL*+ITS+*tufA*+16S. The species number corresponds to the P ID clades in Fig. 3.

Species	Closest Species	Monophyletic?	Intra Dist	Inter Dist - C	Intra/Inter	P ID(Strict)	P ID(Liberal)	Av(MRCA-tips)	P(Randomly	Clade Support	Rosenberg's P(AB)	
1	2	yes	0.005	0.033	0.16	0.87 (0.77, 0.98)	0.96 (0.89, 1.0)	0.0031	NA	4.94E-03		
2	1	yes	0.002	0.033	0.07	0.56 (0.41, 0.71)	0.94 (0.79, 1.0)	0.0011	0.06	NA	4.94E-03	
3	1	yes	0.00E+00	0.064	0.00E+00	0.00E+00 (0.00E+00,	0.96 (0.83, 1.0)	0.00E+00	NA	NA	0.02	
4	5	yes	7.44E-04	0.071	0.01	0.59 (0.44, 0.73)	0.98 (0.83, 1.0)	3.72E-04	NA	0.11		
5	4	yes	0.002	0.071	0.03	0.57 (0.42, 0.72)	0.96 (0.81, 1.0)	0.0011	0.11	NA	0.11	
6	4	yes	0.002	0.229	0.01	0.59 (0.44, 0.74)	0.98 (0.83, 1.0)	0.0011	0.41	NA	9.10E-04	

Supplementary Table 14. Species Delimitation Results of PID for *rbcL+ tufA*. The species number corresponds to the P ID clades in Fig. 4.

Species	Closest Species	Monophyletic?	Intra Dist	Inter Dist -	Intra/Inter	P ID(Strict)	P ID(Liberal)	Av(MRCA-tips)	P(Randomly Distinct)	Clade Supp	Rosenberg's P(AB)
1	2	yes	0.019	0.039	0.49	0.69 (0.59, 0.80)	0.89 (0.83, 0.96)	0.0105	0.09	NA	0.04
2	1	yes	0.00E+00	0.039	0.00E+00	0.00E+00 (0.00E+00, 0.00E+00)	0.96 (0.83, 1.0)	0.00E+00	NA	NA	0.04
3	4	yes	0.00E+00	0.137	0.00E+00	0.00E+00 (0.00E+00, 0.00E+00)	0.96 (0.83, 1.0)	0.00E+00	NA	NA	1
4	2	yes	0.00E+00	0.077	0.00E+00	0.00E+00 (0.00E+00, 0.00E+00)	0.96 (0.83, 1.0)	0.00E+00	NA	NA	1
5	2	yes	0.00E+00	0.08	0.00E+00	0.00E+00 (0.00E+00, 0.00E+00)	0.96 (0.83, 1.0)	0.00E+00	NA	NA	0.01
6	5	yes	0.001	0.2	0.01	0.87 (0.72, 1.0)	0.98 (0.87, 1.0)	0.0012	NA	4.08E-03	

Supplementary Table 15. Species Delimitation Results of PID for ITS+16S. The species number corresponds to the P ID clades in Fig. 5.

Species	Closest Species	Monophyletic?	Intra Dist	Inter Dist - Closest	Intra/Inter	P ID(Strict)	P ID(Liberal)	Av(MRCA-tips)	P(Randomly Distinct)	Clade Support	Rosenberg's P(AB)
1	3	yes	8.65E-04	0.06	0.01	0.98 (0.92, 1.0)	1.00 (0.96, 1.0)	4.45E-04	NA	9.10E-04	
2	1	yes	0.004	0.064	0.07	0.56 (0.41, 0.71)	0.94 (0.79, 1.0)	0.0022	0.93	NA	9.10E-04
3	1	yes	0.023	0.06	0.39	0.53 (0.35, 0.71)	0.78 (0.64, 0.93)	0.0119	1	NA	9.20E-05
4	8	yes	0.001	0.027	0.05	0.56 (0.41, 0.71)	0.95 (0.80, 1.0)	7.17E-04	0.99	NA	2.75E-03
5	6	yes	0.00E+00	0.007	0.00E+00	0.59 (0.44, 0.74)	0.98 (0.83, 1.0)	0.00E+00	NA	NA	0.11
6	5	yes	0.004	0.007	0.6	0.29 (0.13, 0.44)	0.61 (0.46, 0.77)	0.0022	NA	0.11	
7	8	yes	0.00E+00	0.012	0.00E+00	0.59 (0.44, 0.74)	0.98 (0.83, 1.0)	0.00E+00	NA	NA	0.03
8	5	yes	0.002	0.011	0.2	0.73 (0.59, 0.88)	0.93 (0.82, 1.0)	0.0012	0.99	NA	0.03

Supplementary Table 16**.** Primer sequences and annealing temperatures used to amplify the different regions. Names of the forward (-F) and reverse (-R) primers pairs are indicated and also their references.

**Name**	**Sequence 5'–3'**	**Annealing temperature (°C)**	**Source**
rbcL			
rbcL-F	ATG KCT CCA CAA ACT GAA ACT A	49-51	Sun et al. 2009
rbcL-R	TTA AAG WGT ATC GAT WGT TTC GA	49-51	Sun et al. 2009
*RcbL*Z-F	CAA CCA GGT GTT CCA SCT GAA G	49-50	This study
*RcbL*Z-R	CTA AAG CTG GCA TGT GCC ATA C	49-50	This study
**16S**			
359F-T	GGG GAA TTT TCC GCA ATG GG	47-50	Burja et al. 2001
781R(b)	GAC TAC AGG GGT ATC TAA TCC CTT T	47-50	Burja et al. 2001
16SZ-R	GGT ATC TWA TCC CTT TYG CT	47-50	This study
16SZ-F	CGC AAT GGG CGA AAG CCT G	47-50	This study
**ITS**			
NS7m-F	GGC AAT AAC AGG TCT GT	56	Bock et al. 2011
LR1850-R	CCT CAC GGT ACT TGT TC	56	Bock et al. 2011
tufA			
tufA-F	TGA AAC AGA AMA WCG TCA TTA TGC	47-53	Famà et al. 2002
tufA-R	CCT TCN CGA ATM GCR AAW CGC	47-53	Famà et al. 2002
*tufA-S*F	TGG ATG GTG CWA TTY TWG	47-50	This study
*tufA-S*R	GGT TTT GCW AAA ACC ATW CCA CG	47-50	This study

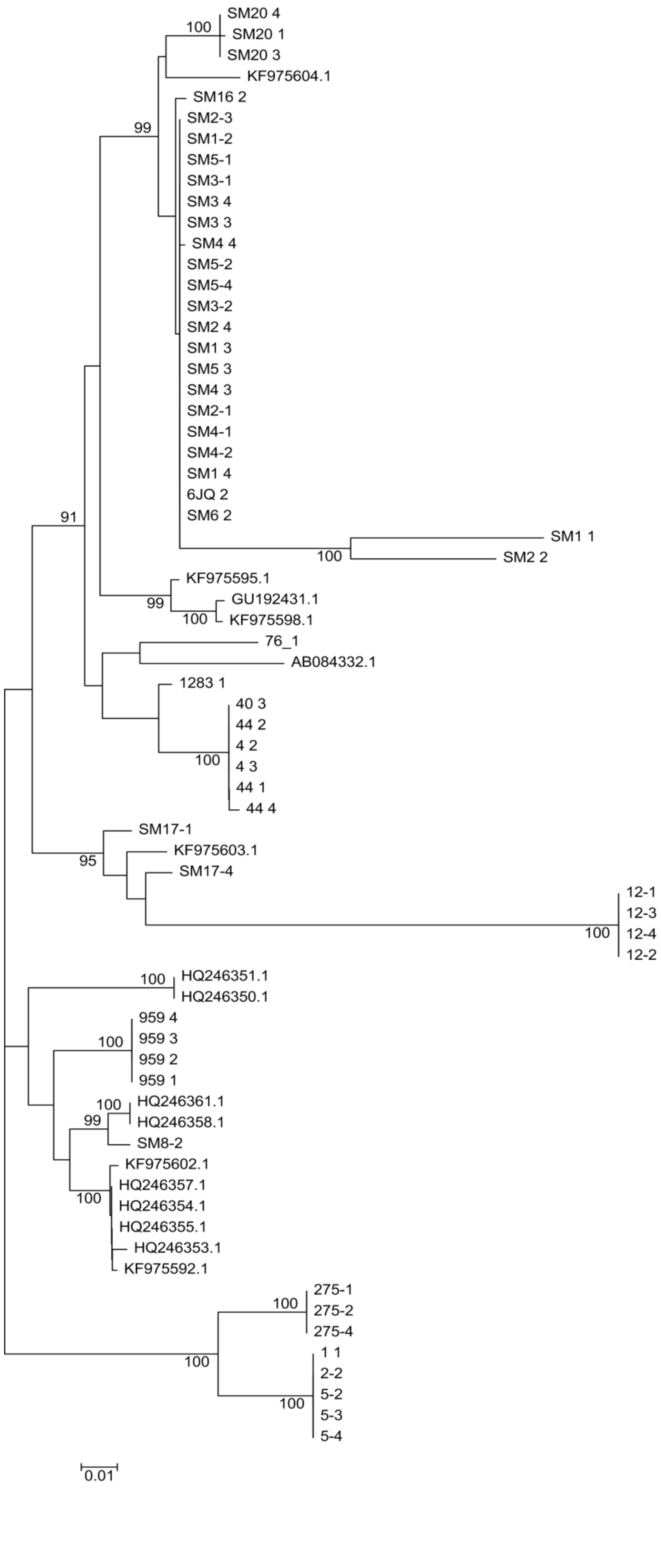

Supplementary Fig. 1. Neighbor joining tree for the *rbcL*

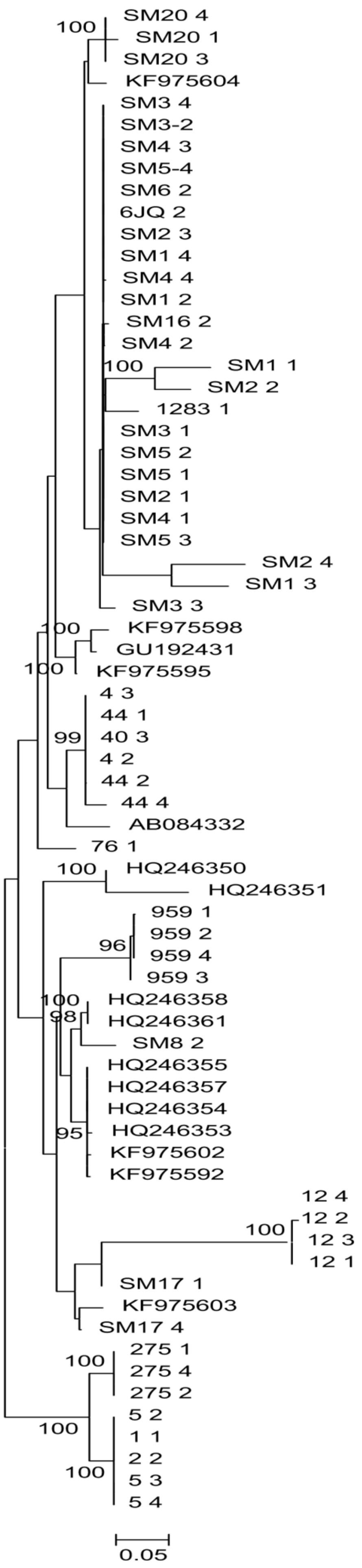

Supplementary Fig. 2. Maximum likelihood tree for the *rbcL* gene.

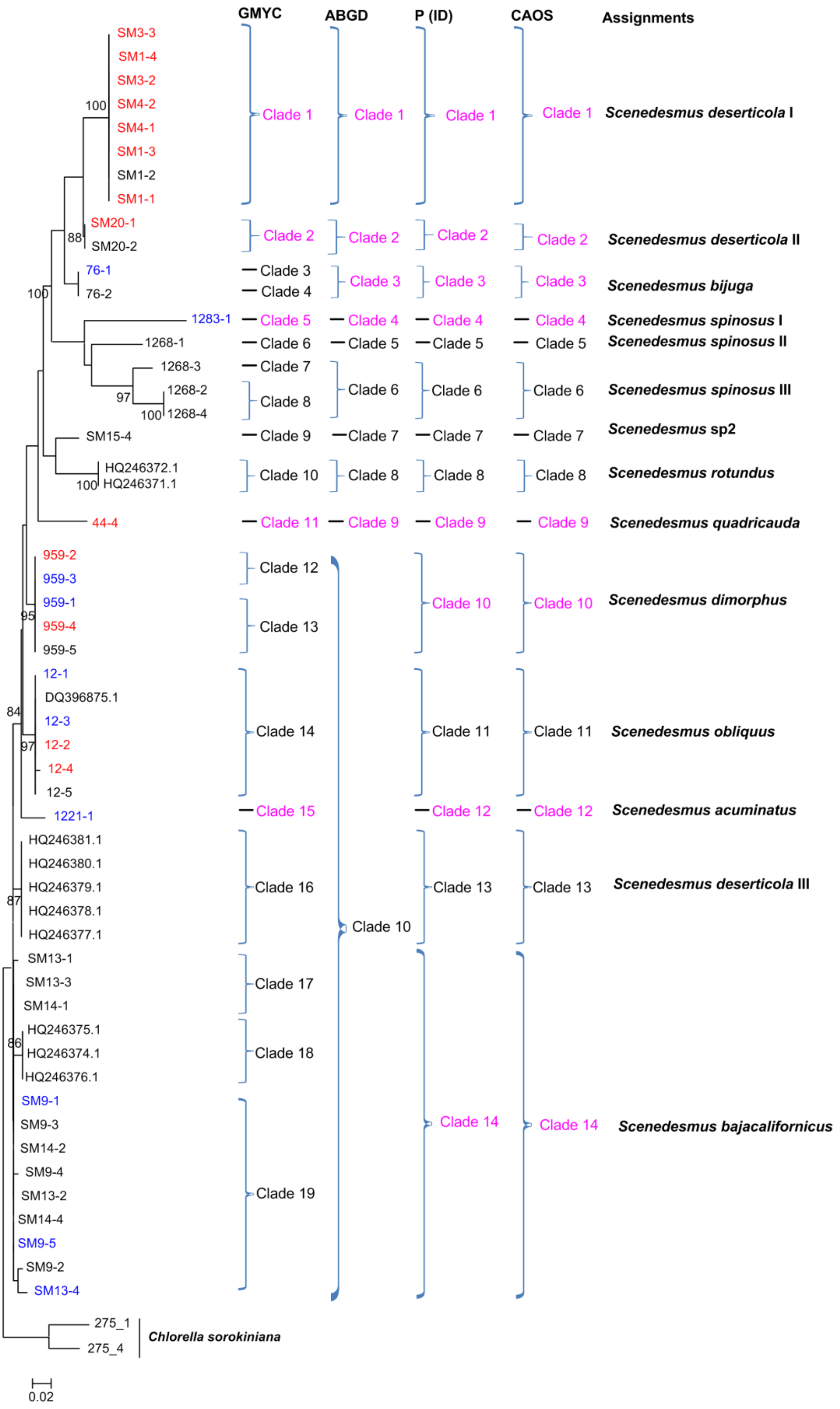
GMYC
ABGD
P (ID)
CAOS
Assignments
SM3-3
SM1-4
SM3-2
SM4-2
SM4-1
SM1-3
SM1-2
SM1-1
SM20-1
SM20-2
76-1
76-2
1283-1
1268-1
1268-3
1268-2
1268-4
SM15-4
HQ246372.1
HQ246371.1
44-4
959-2
959-3
959-1
959-4
959-5
12-1
DQ396875.1
12-3
12-2
12-4
12-5
1221-1
HQ246381.1
HQ246380.1
HQ246379.1
HQ246378.1
HQ246377.1
SM13-1
SM13-3
SM14-1
HQ246375.1
HQ246374.1
HQ246376.1
SM9-1
SM9-3
SM14-2
SM9-4
SM13-2
SM14-4
SM9-5
SM9-2
SM13-4
275_1
275_4
Chlorella sorokiniana
0.02
Scenedesmus deserticola I
Scenedesmus deserticola II
Scenedesmus bijuga
Scenedesmus spinosus I
Scenedesmus spinosus II
Scenedesmus spinosus III
Scenedesmus sp2
Scenedesmus rotundus
Scenedesmus quadricauda
Scenedesmus dimorphus
Scenedesmus obliquus
Scenedesmus acuminatus
Scenedesmus deserticola III
Scenedesmus bajacalifornicus

Supplementary Fig. 3. Bayesian phylogenetic tree for the *tufA* gene. Vertical brace on the right indicate the clades detected by the tree-based GMYC, PID and the distance-based ABGD approach, the character-based CAOS and the taxa assignment. The clades highlighted in pink were also detected by *rbcL*, 16S and ITS gene loci. For samples colored in red, 16S, *rbcL* and ITS sequences were also available. For samples colored in blue, two of 16S, *rbcL* and ITS sequences were available.

Supplementary Fig. 4. Neighbor joining tree for the *tufA* gene.

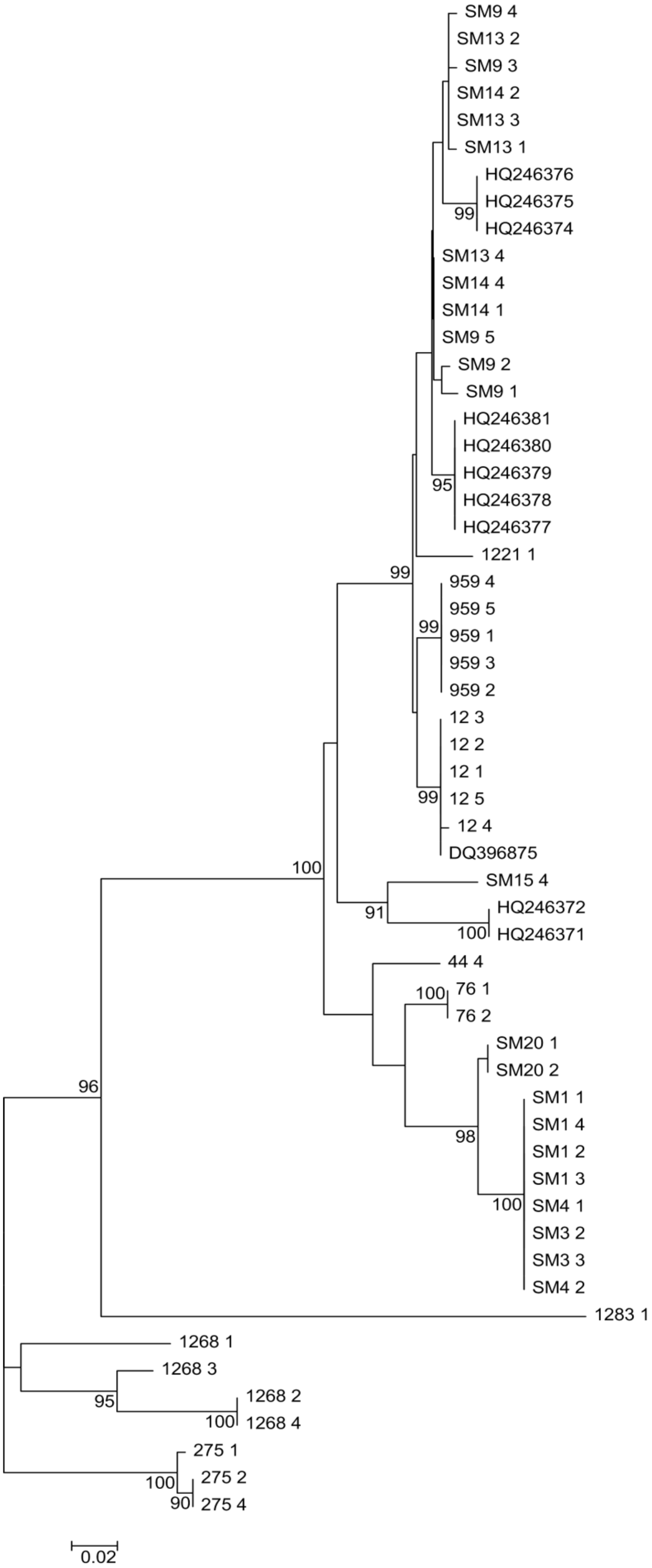

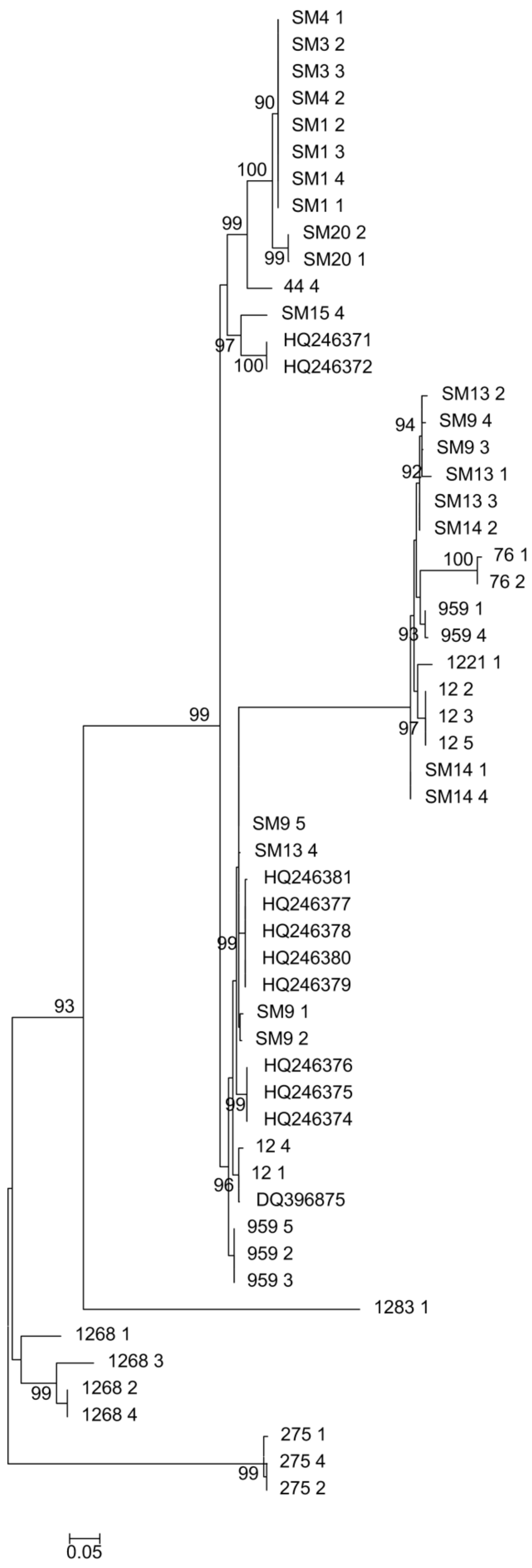

Supplementary Fig. 5. Maximum likelihood tree for the *tufA* gene.

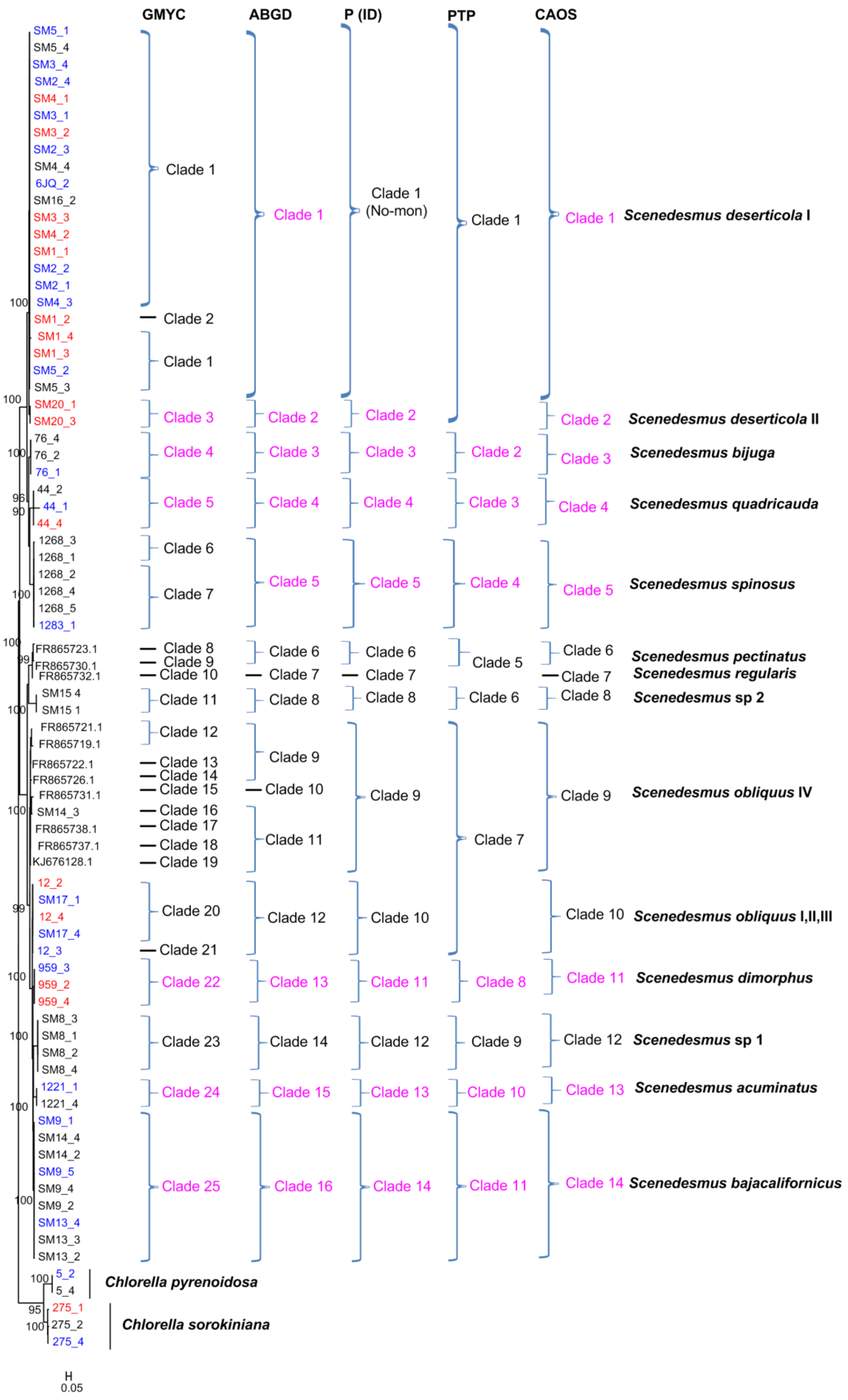
GMYC
ABGD
P (ID)
PTP
CAOS
SM5_1
SM5_4
SM3_4
SM2_4
SM4_1
SM3_1
SM3_2
SM2_3
SM4_4
6JQ_2
SM16_2
SM3_3
SM4_2
SM1_1
SM2_2
SM2_1
SM4_3
SM1_2
SM1_4
SM1_3
SM5_2
SM5_3
SM20_1
SM20_3
76_4
76_2
76_1
44_2
44_1
44_4
1268_3
1268_1
1268_2
1268_4
1268_5
1283_1
FR865723.1
FR865730.1
FR865732.1
SM15 4
SM15 1
FR865721.1
FR865719.1
FR865722.1
FR865726.1
FR865731.1
SM14_3
FR865738.1
FR865737.1
KJ676128.1
12_2
SM17_1
12_4
SM17_4
12_3
959_3
959_2
959_4
SM8_3
SM8_1
SM8_2
SM8_4
1221_1
1221_4
SM9_1
SM14_4
SM14_2
SM9_5
SM9_4
SM9_2
SM13_4
SM13_3
SM13_2
5_2
5_4
275_1
275_2
275_4
100
100
100
96
90
100
100
99
100
100
99
100
100
100
100
100
95
100
Clade 1
Clade 2
Clade 1
Clade 3
Clade 4
Clade 5
Clade 6
Clade 7
Clade 8
Clade 9
Clade 10
Clade 11
Clade 12
Clade 13
Clade 14
Clade 15
Clade 16
Clade 17
Clade 18
Clade 19
Clade 20
Clade 21
Clade 22
Clade 23
Clade 24
Clade 25
Clade 1
Clade 2
Clade 3
Clade 4
Clade 5
Clade 6
Clade 7
Clade 8
Clade 9
Clade 10
Clade 11
Clade 12
Clade 13
Clade 14
Clade 15
Clade 16
Clade 1 (No-mon)
Clade 2
Clade 3
Clade 4
Clade 5
Clade 6
Clade 7
Clade 8
Clade 9
Clade 10
Clade 11
Clade 12
Clade 13
Clade 14
Clade 1
Clade 2
Clade 3
Clade 4
Clade 5
Clade 6
Clade 7
Clade 8
Clade 9
Clade 10
Clade 11
Clade 1
Clade 2
Clade 3
Clade 4
Clade 5
Clade 6
Clade 7
Clade 8
Clade 9
Clade 10
Clade 11
Clade 12
Clade 13
Clade 14
Scenedesmus deserticola I
Scenedesmus deserticola II
Scenedesmus bijuga
Scenedesmus quadricauda
Scenedesmus spinosus
Scenedesmus pectinatus
Scenedesmus regularis
Scenedesmus sp 2
Scenedesmus obliquus IV
Scenedesmus obliquus I,II,III
Scenedesmus dimorphus
Scenedesmus sp 1
Scenedesmus acuminatus
Scenedesmus bajacalifornicus
Chlorella pyrenoidosa
Chlorella sorokiniana
0.05

Supplementary Fig. 6. Bayesian phylogenetic tree for the ITS gene. Vertical brace on the right indicate the clades detected by the tree-based GMYC, PID, PTP and the distance-based ABGD approach, the character-based CAOS and the taxal assignment. The clades highlighted in pink were also detected by *rbcL*, 16S and *tufA* gene loci. For samples colored in red, 16S, *rbcL* and *tufA* sequences were also available. For samples colored in blue, two of 16S, *rbcL* and *tufA* sequences were available.

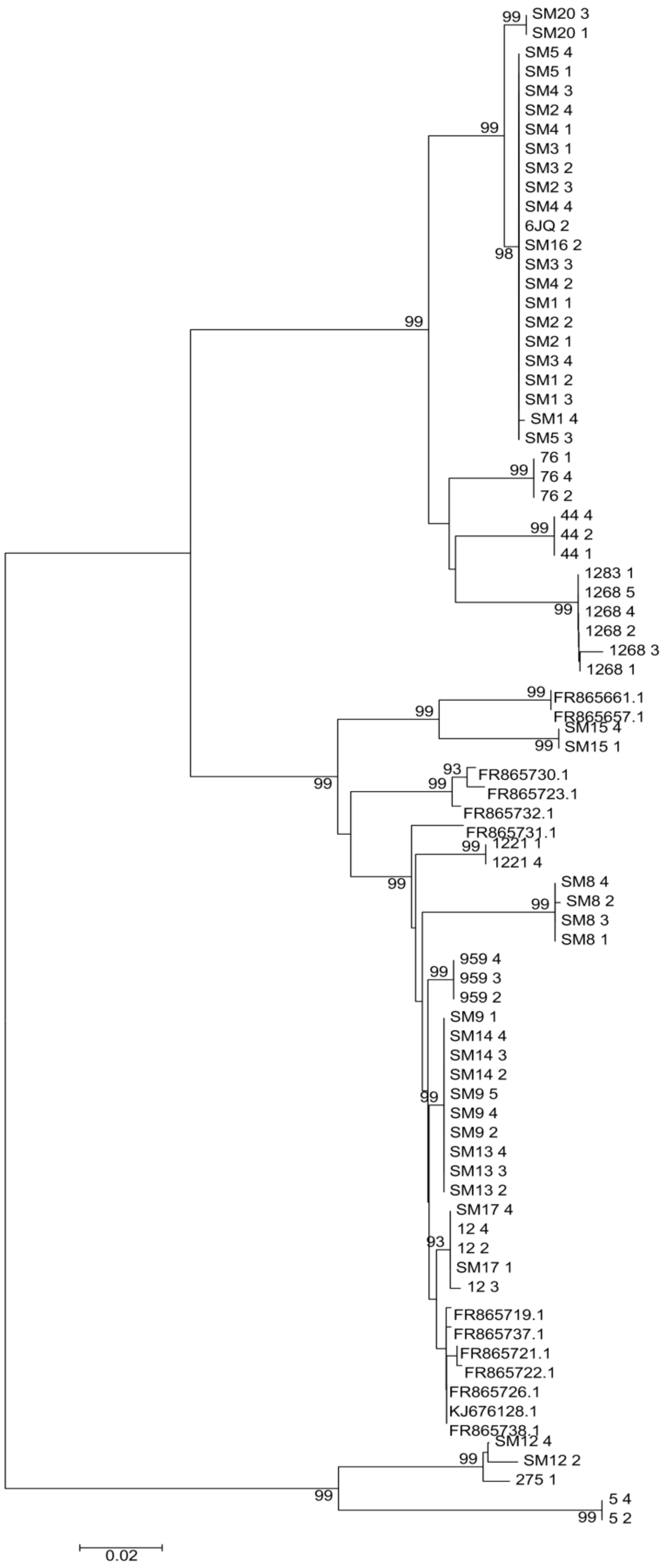

Supplementary Fig. 7. Neighbor joining tree for the ITS gene.

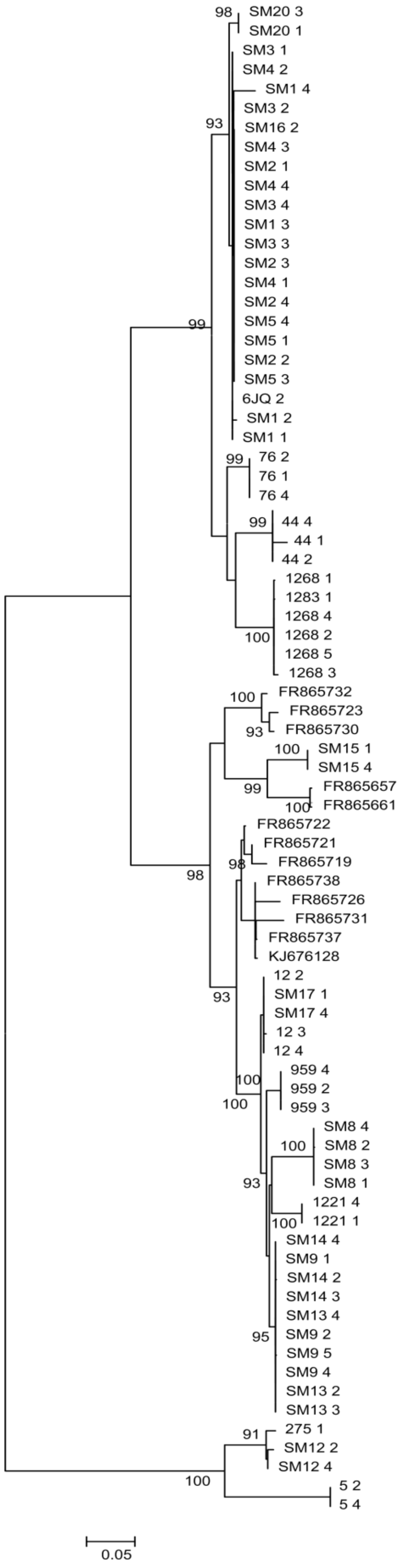

Supplementary Fig. 8. Maximum likelihood tree for the ITS gene.

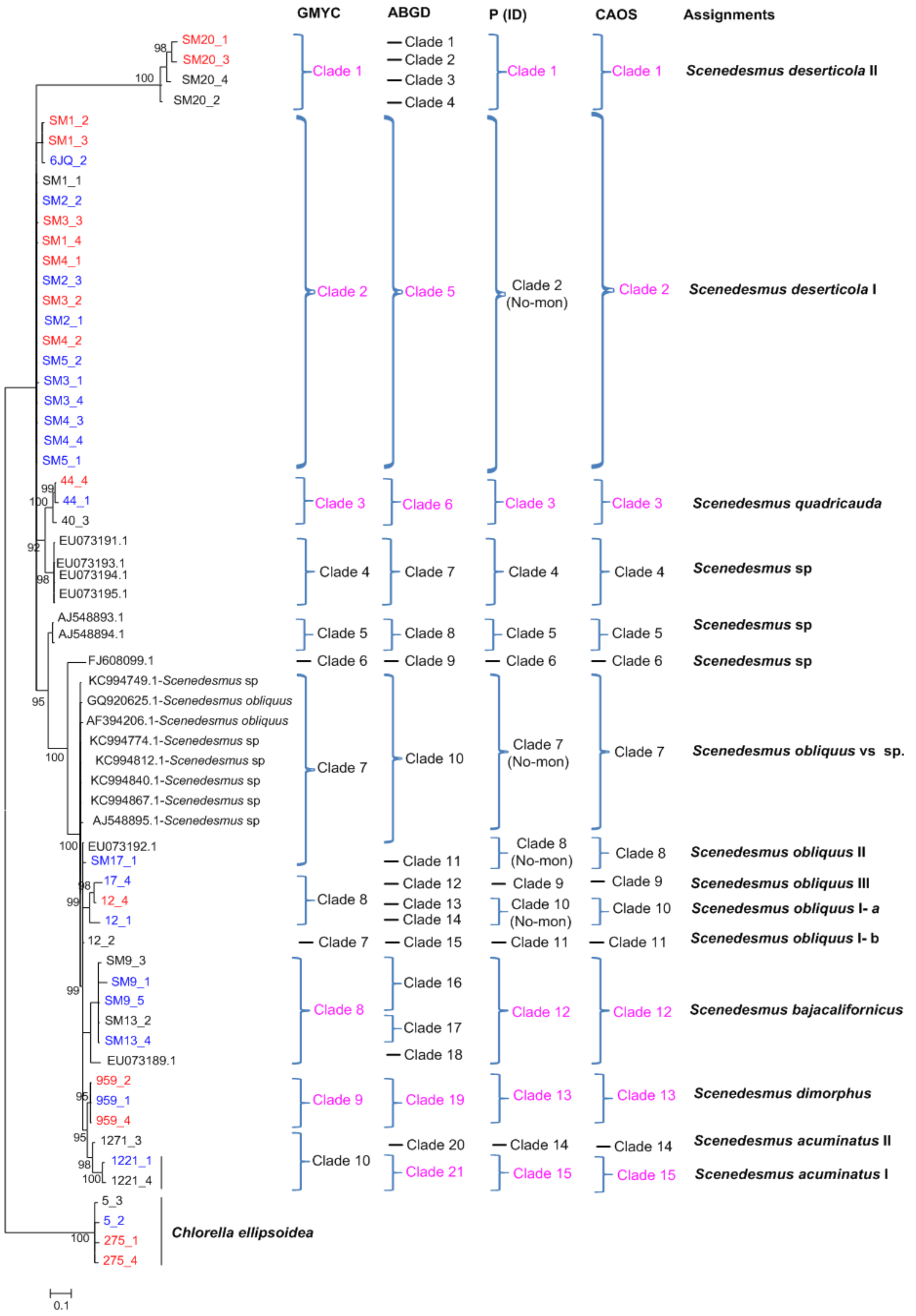

Supplementary Fig. 9. Bayesian phylogenetic tree for the16S gene. Vertical brace on the right indicate the clades detected by the tree-based GMYC, PID, the distance-based ABGD approach, the character-based CAOS and the final assignment. The clades highlighted in pink were also detected by *rbcL*, 16S and *tufA* gene loci. For samples colored in red, 16S, *rbcL* and *tufA* sequences were also available. For samples colored in blue, two of 16S, *rbcL* and *tufA* sequences were available.

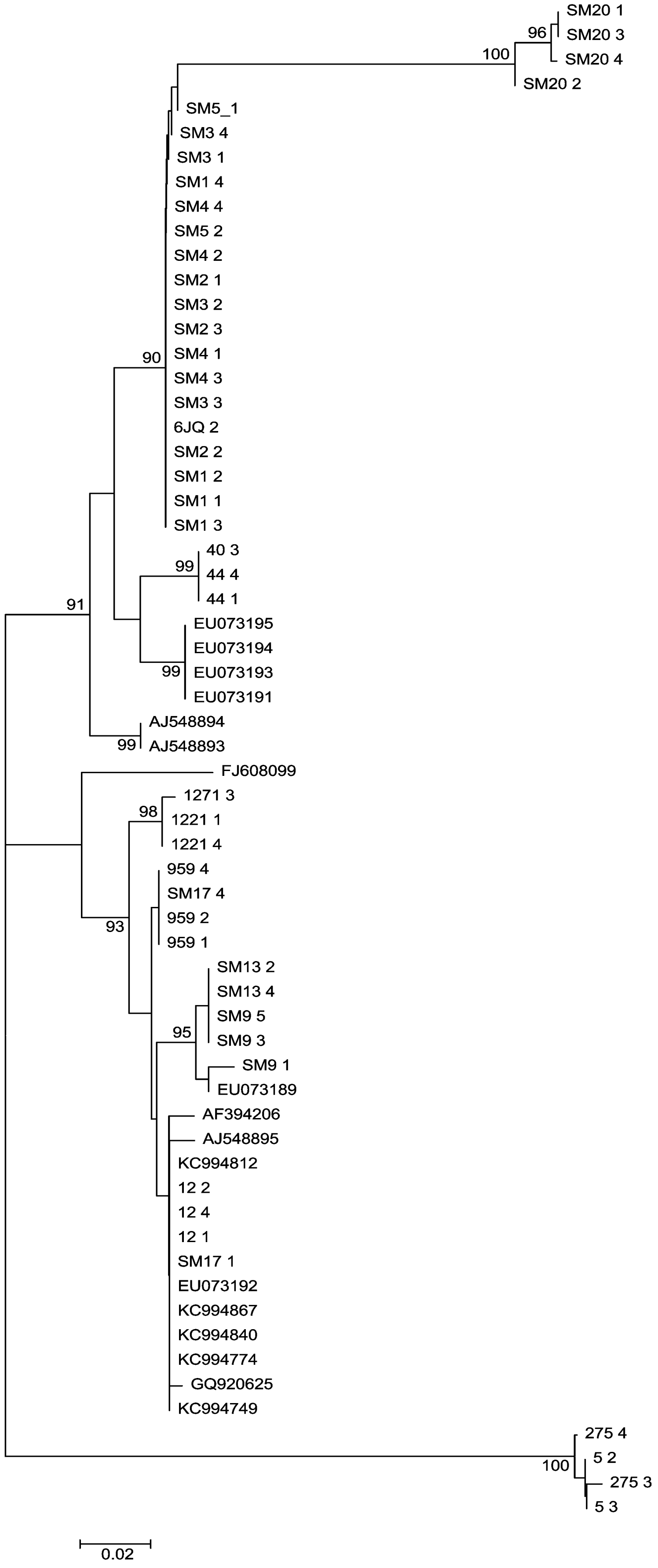

Supplementary Fig. 10. Neighbor joining tree for the 16S gene.

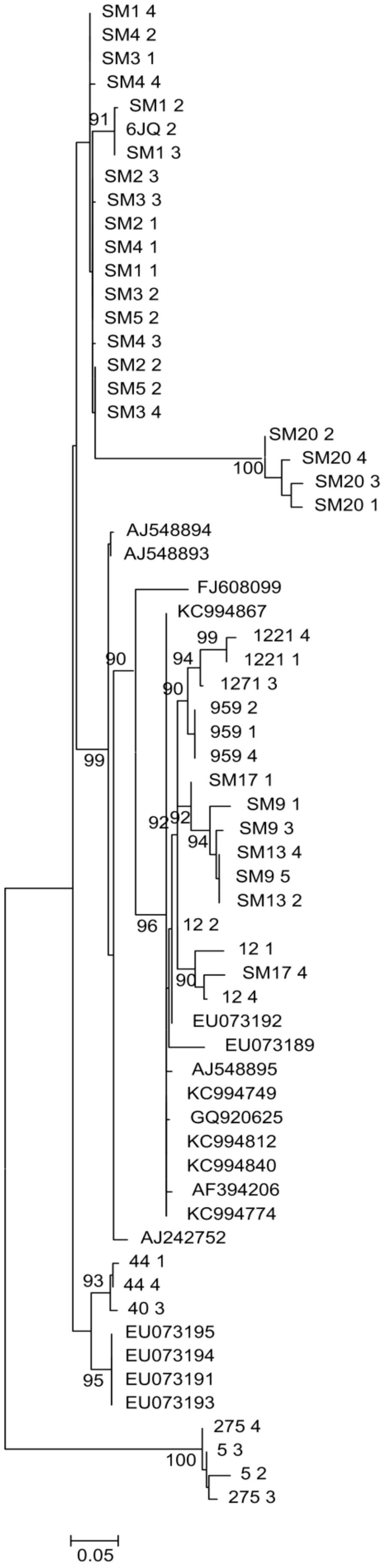

Supplementary Fig. 11. Maximum likelihood tree for the 16S gene.

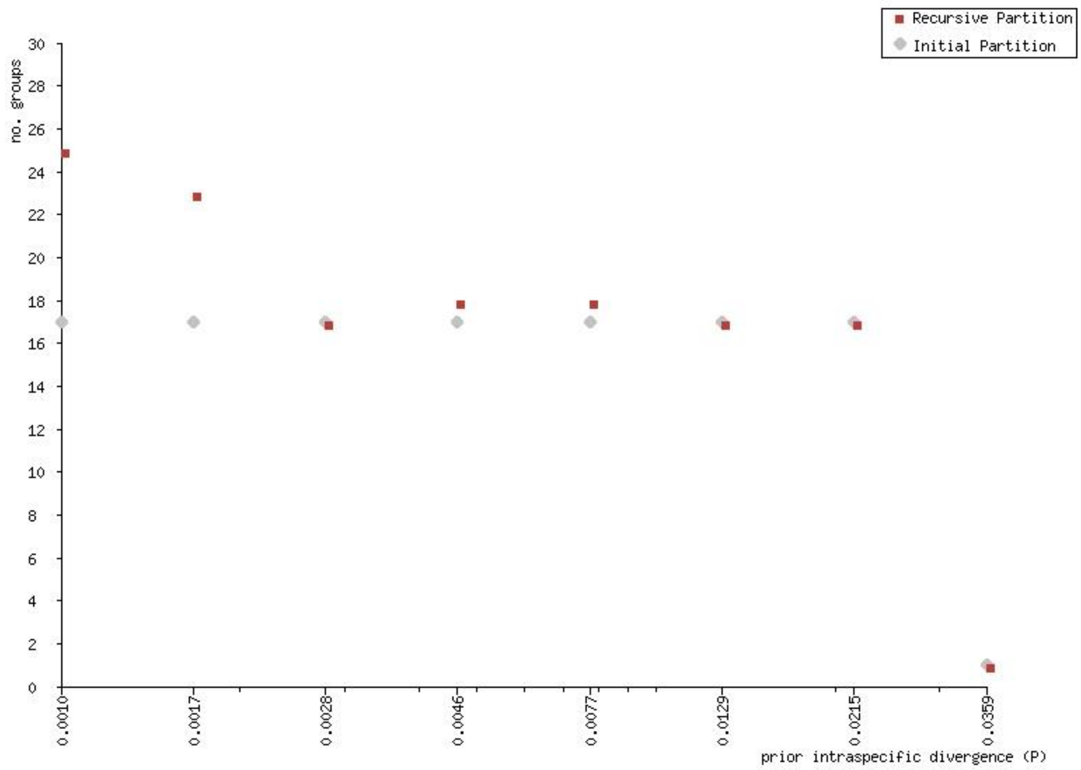

Supplementary Fig. 12. Automatic partition of tellinaceans based on *rbcL* gene. The number of groups inside the partition (initial and recursive) of each given prior intraspecific divergence value were reported.

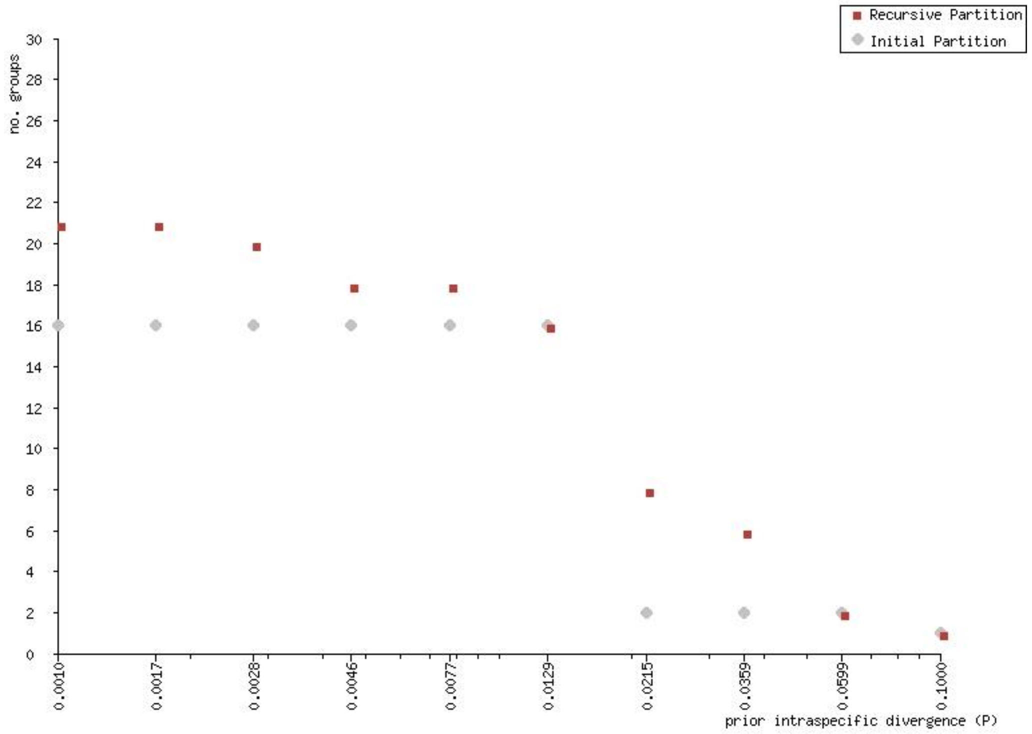

Supplementary Fig. 13. Automatic partition of tellinaceans based on ITS gene. The number of groups inside the partition (initial and recursive) of each given prior intraspecific divergence value were reported.

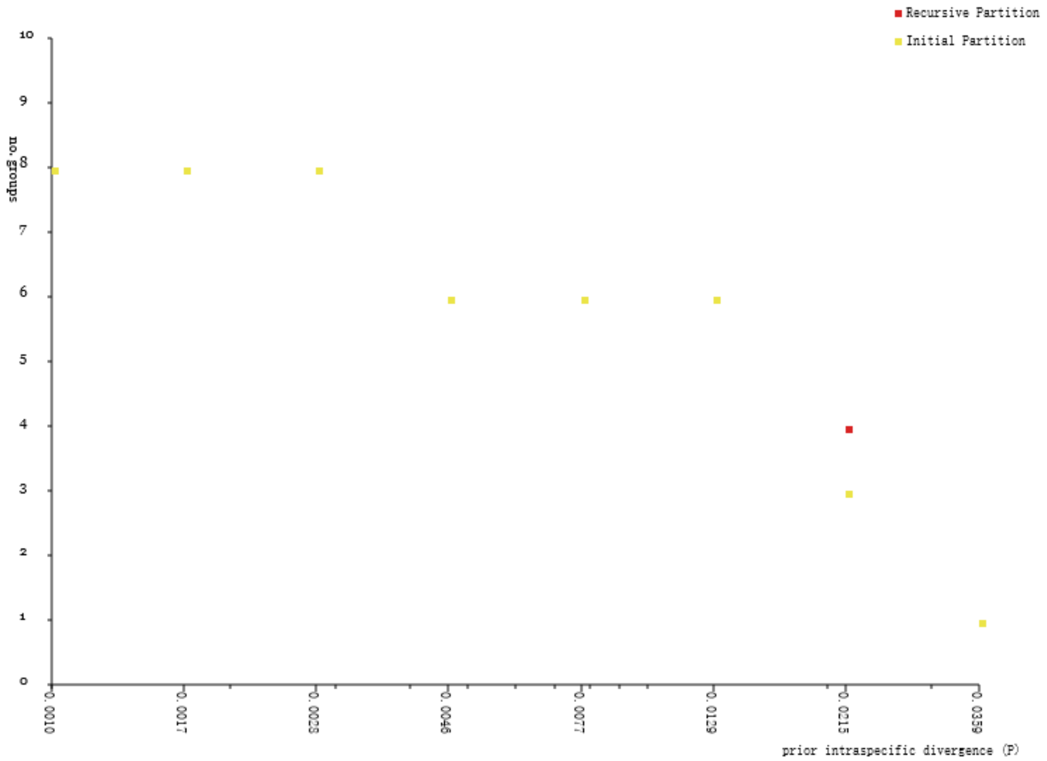

Supplementary Fig. 14. Automatic partition of tellinaceans based on 16S gene. The number of groups inside the partition (initial and recursive) of each given prior intraspecific divergence value were reported.

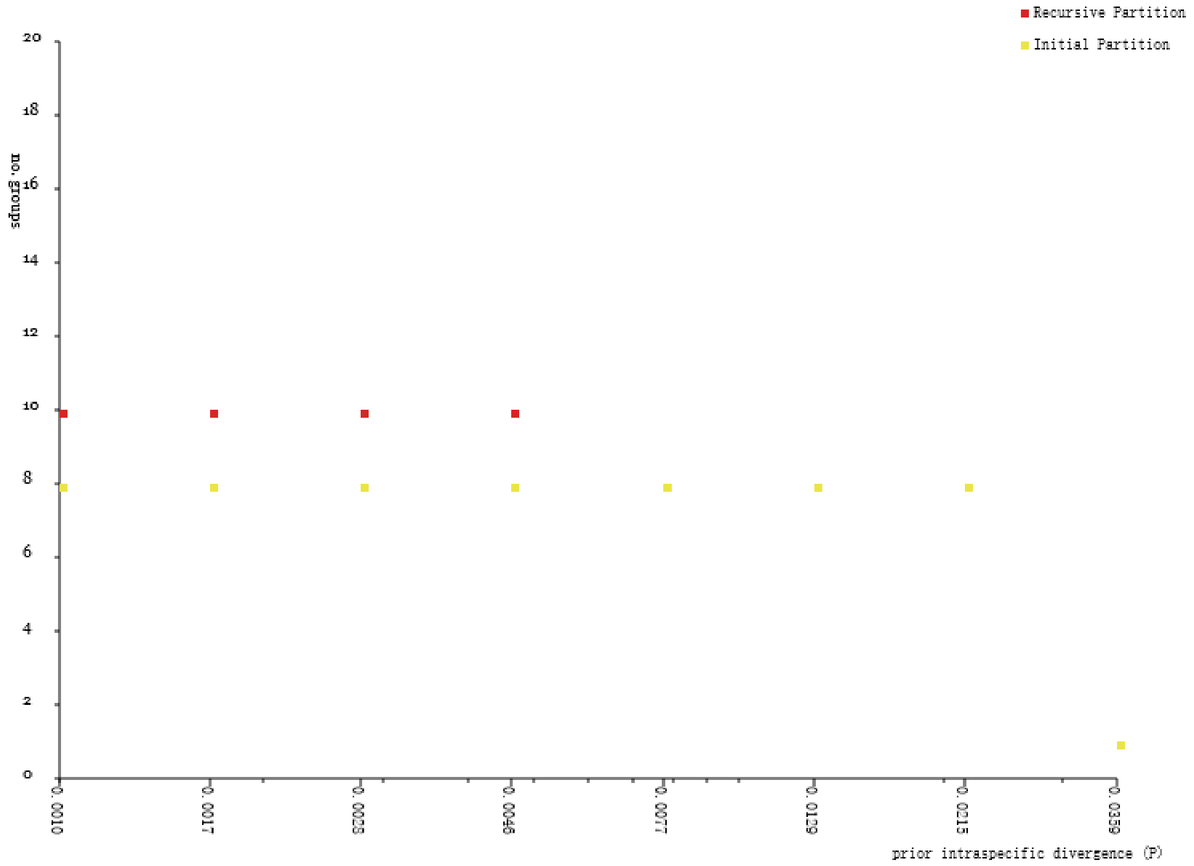

Supplementary Fig. 15. Automatic partition of tellinaceans based on *tufA* gene. The number of groups inside the partition (initial and recursive) of each given prior intraspecific divergence value were reported.

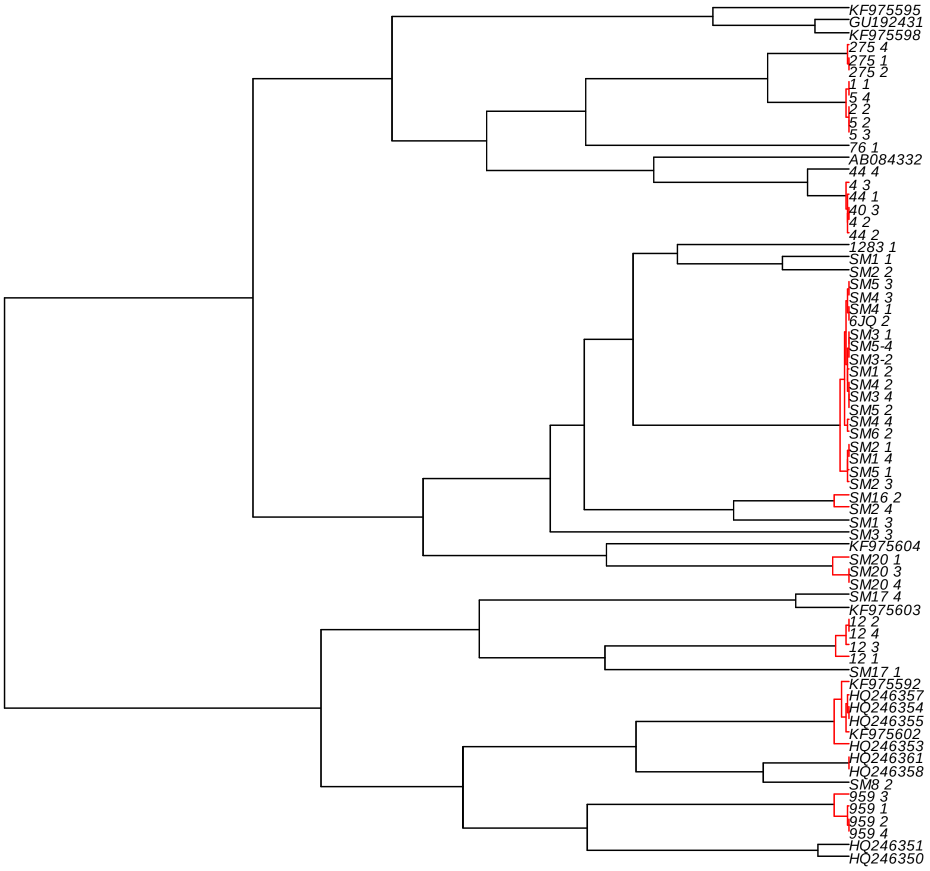

Supplementary Fig. 16. Genetic groups recovered by GMYC model for *rbcL*.

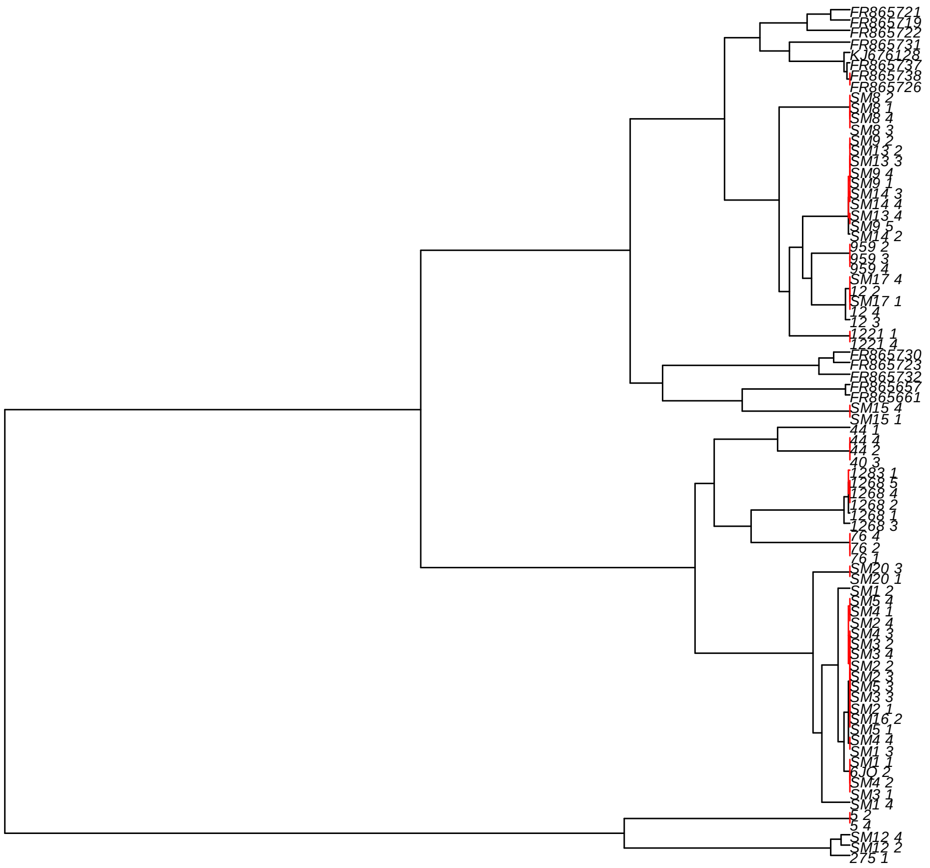

Supplementary Fig. 17. Genetic groups recovered by GMYC model for ITS.

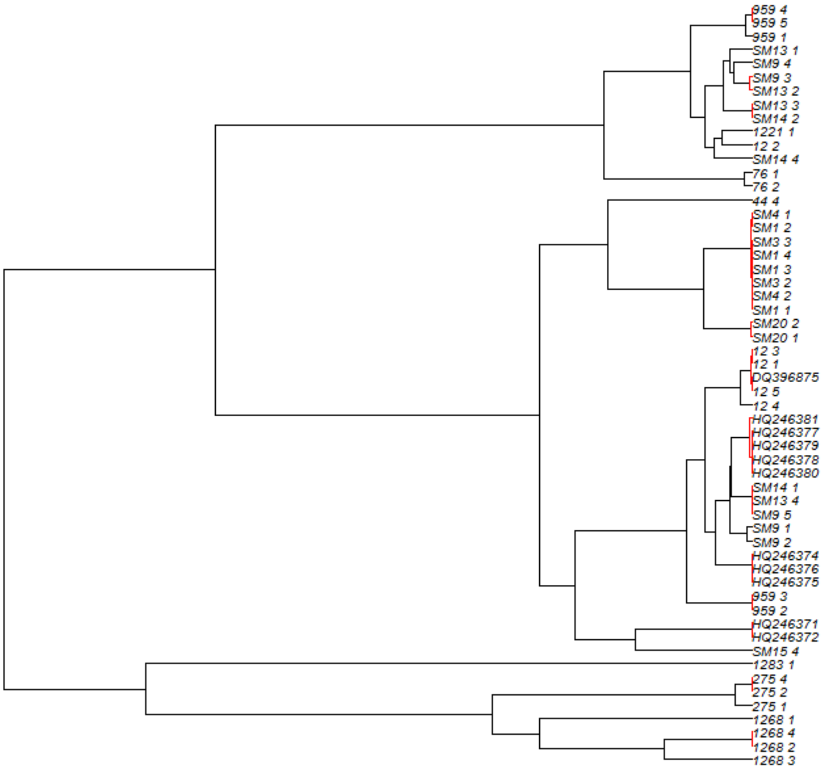

Supplementary Fig. 18. Genetic groups recovered by GMYC model for *tufA*.

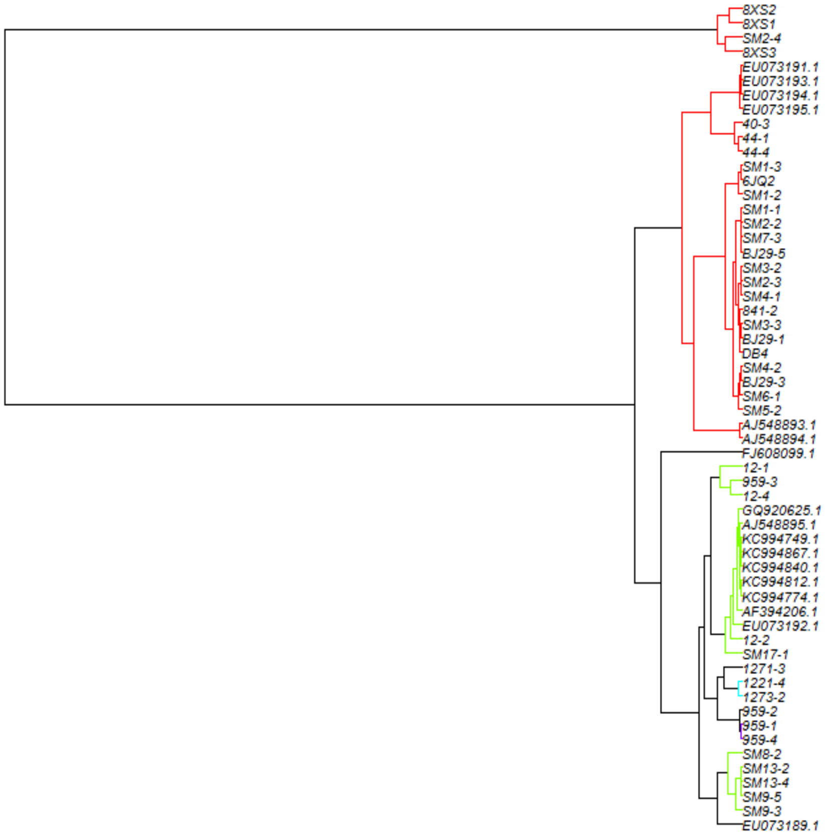

Supplementary Fig. 19. Genetic groups recovered by GMYC model for 16S.

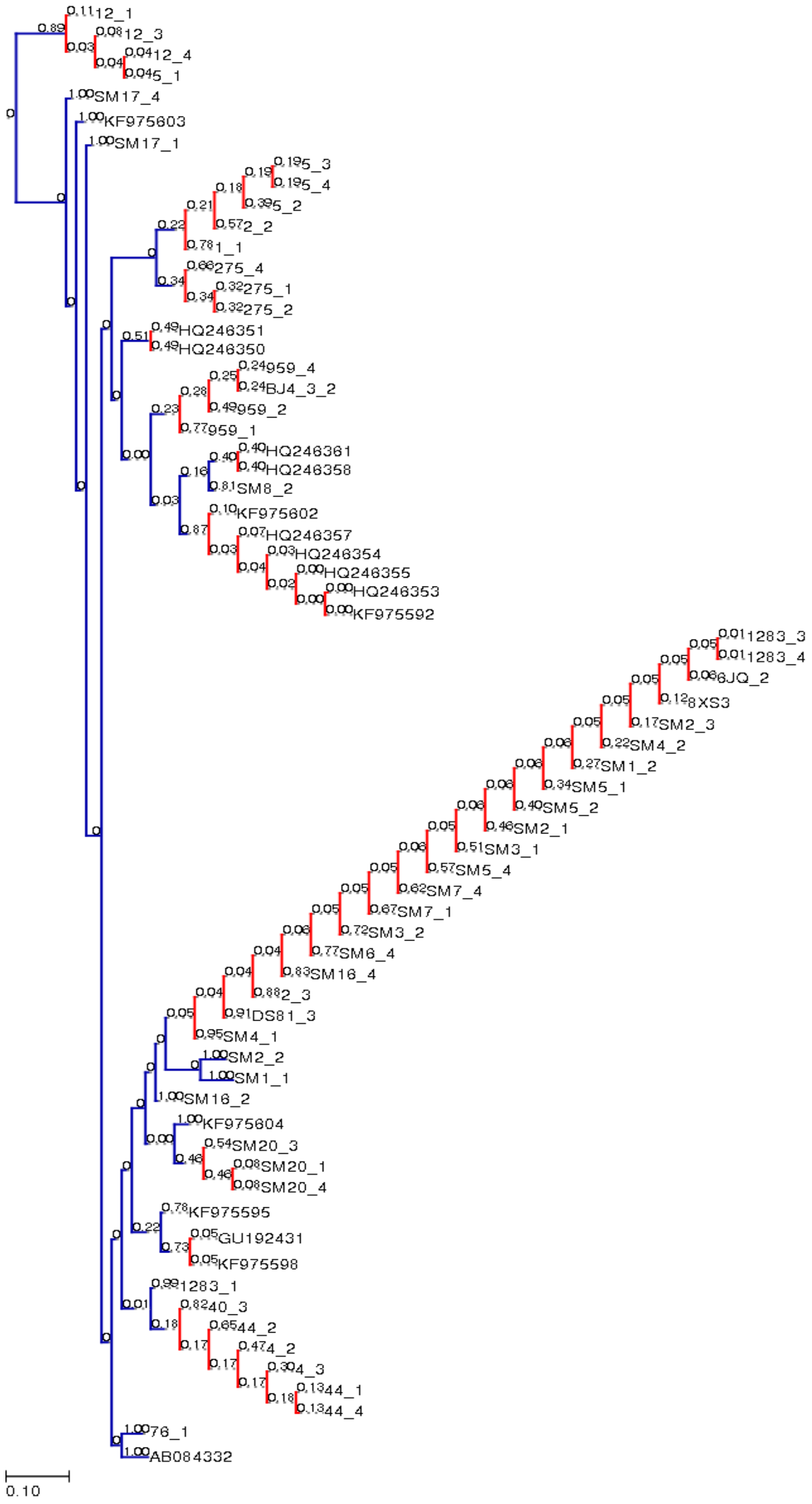

Supplementary Fig. 20. Genetic groups recovered by dPTP analysis for *rbcL*.

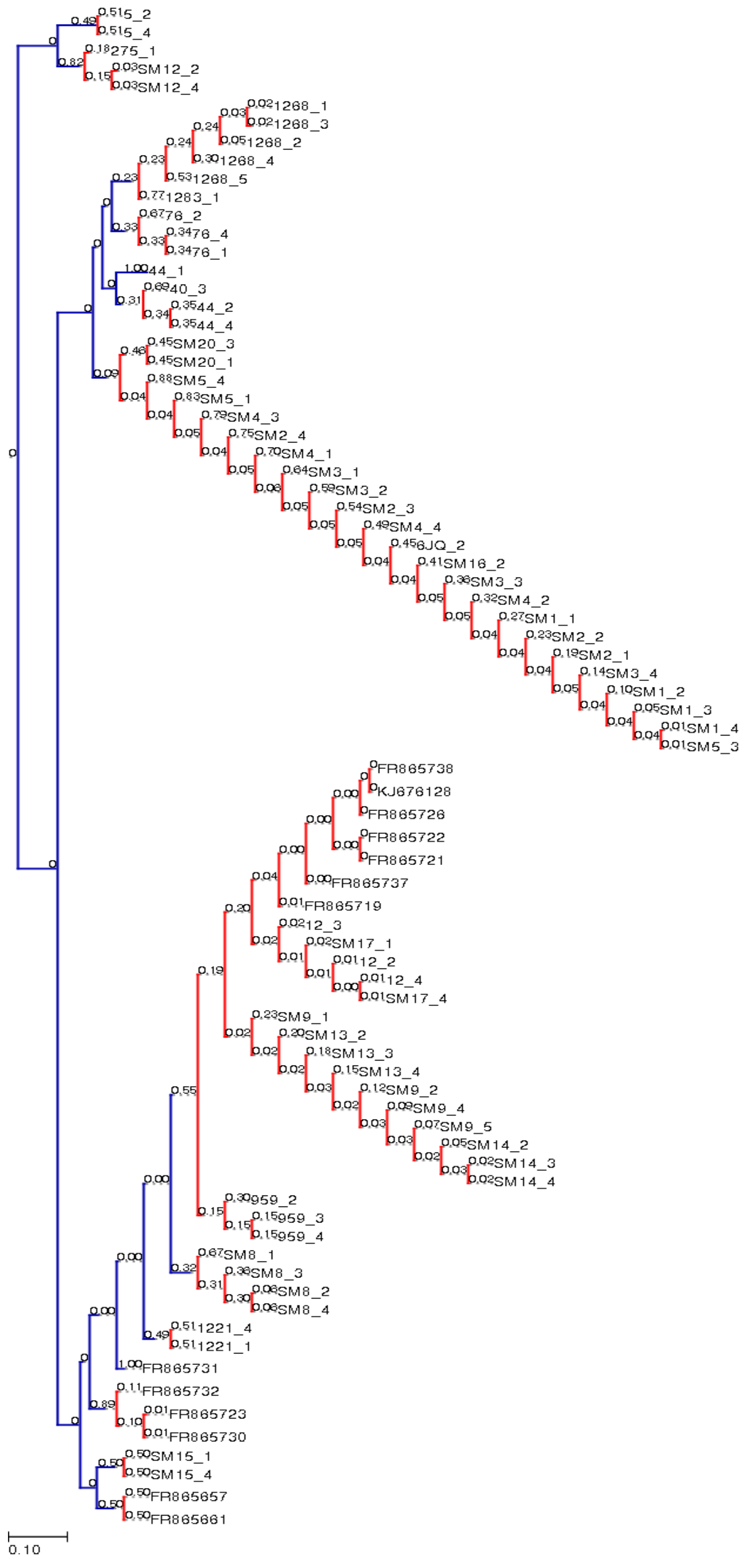

Supplementary Fig. 21. Genetic groups recovered by dPTP analysis for ITS.

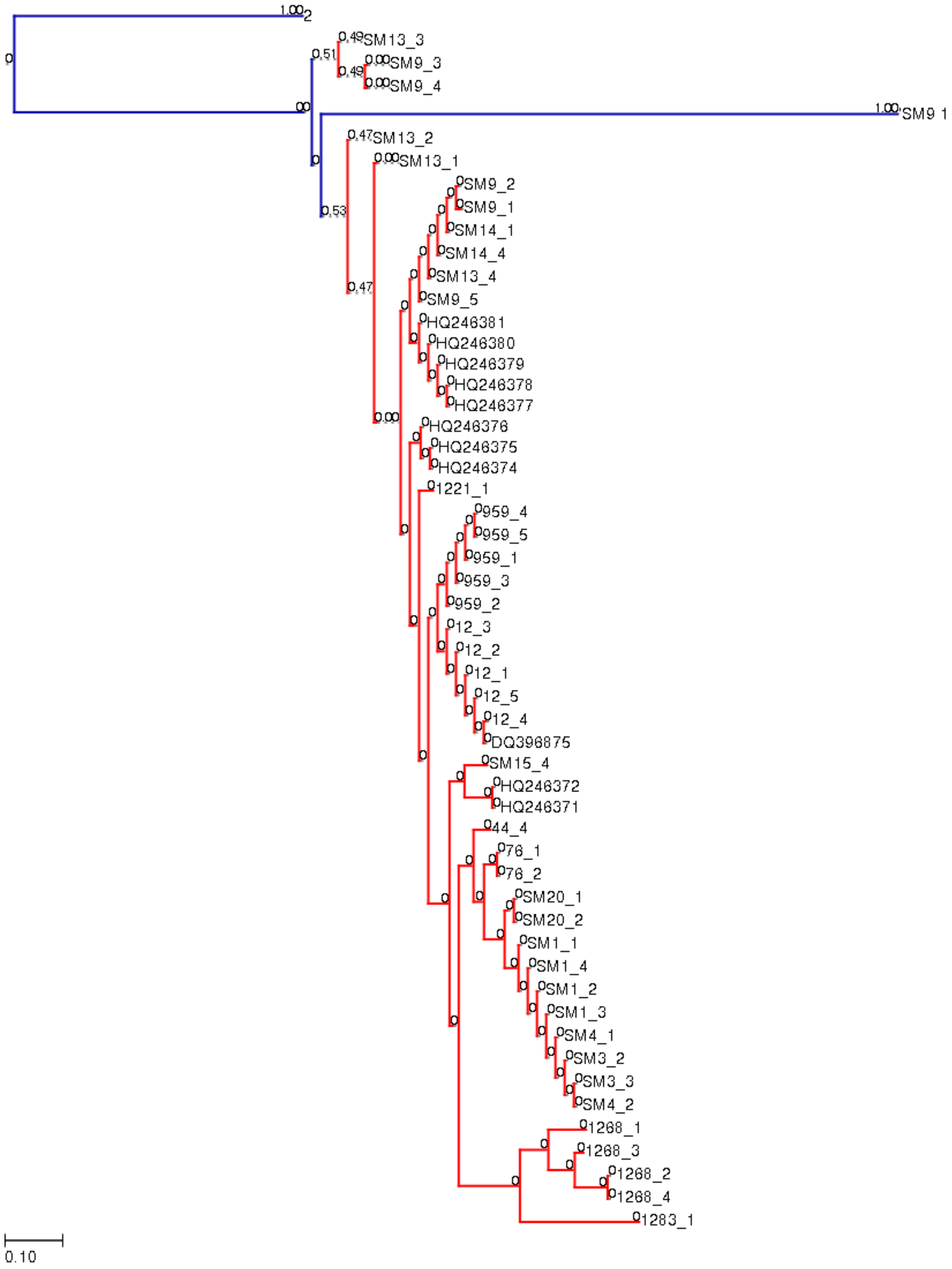

Supplementary Fig. 22. Genetic groups recovered by dPTP analysis for *tufA*.

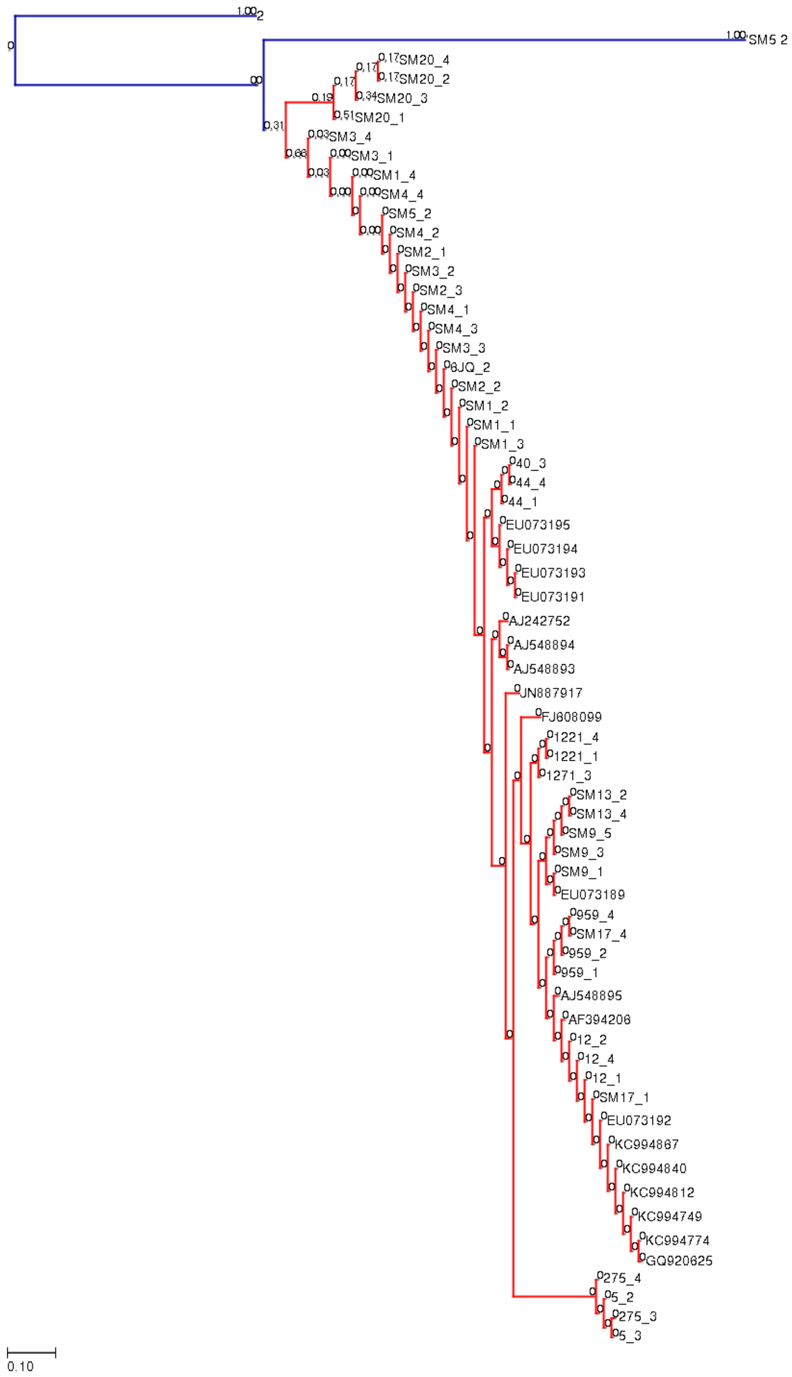

Supplementary Fig. 23. Genetic groups recovered by dPTP analysis for 16S.

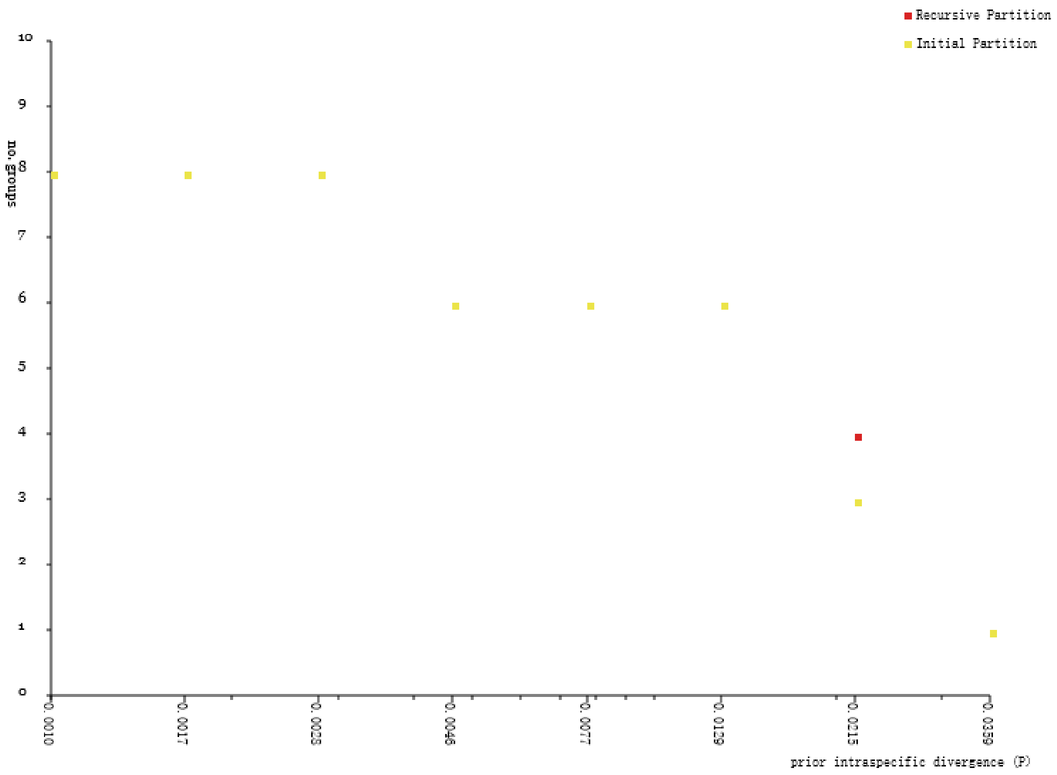

Supplementary Fig. 24. Automatic partition of tellinaceans based on *rbcL*+ITS+16S+*tufA* sequences. The number of groups inside the partition (initial and recursive) of each given prior intraspecific divergence value were reported.

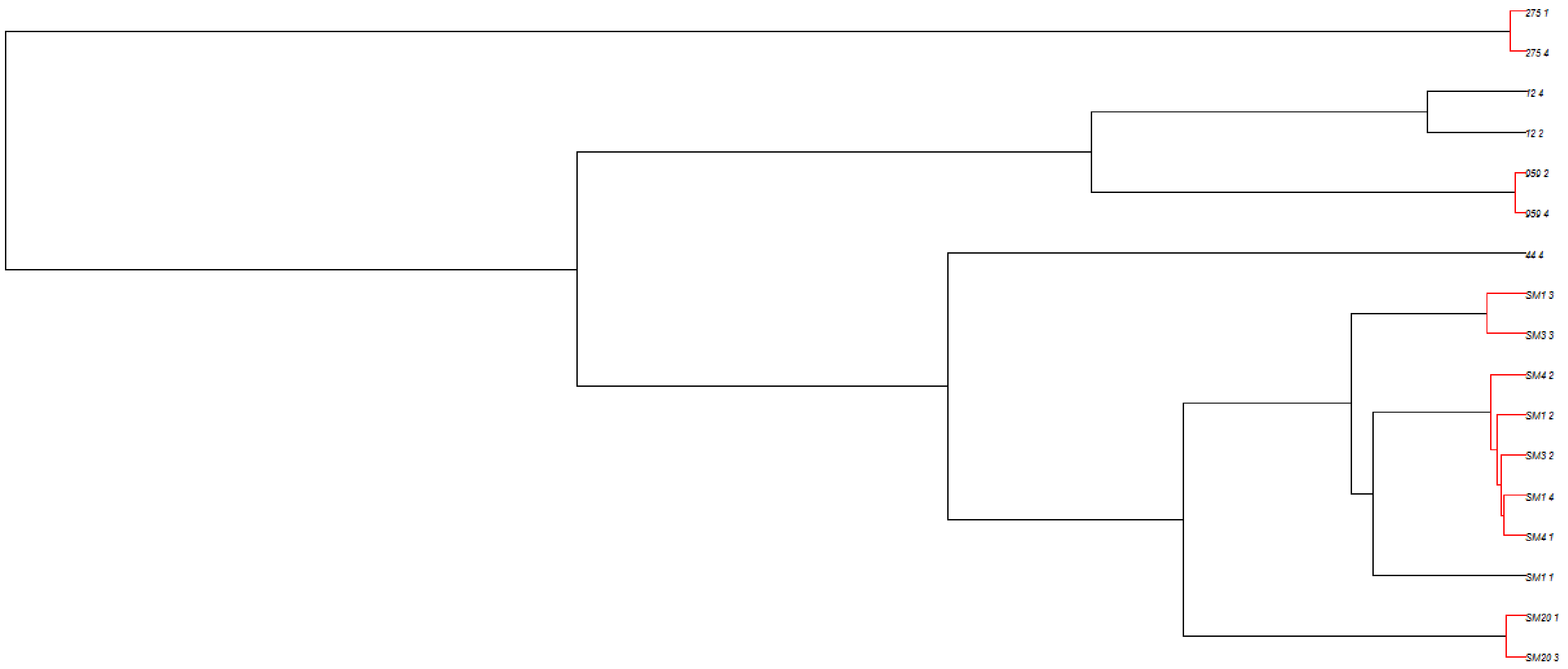

Supplementary Fig. 25. Genetic groups recovered by GMYC model for *rbcL*+ITS+16S+*tufA*.

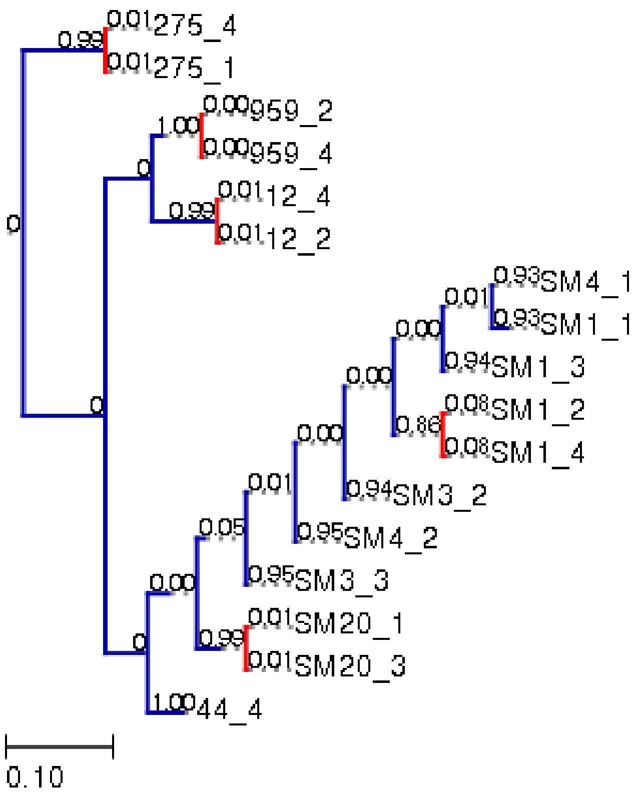

Supplementary Fig. 26. Genetic groups recovered by dPTP analysis for *rbcL*+ITS+16S+*tufA*.

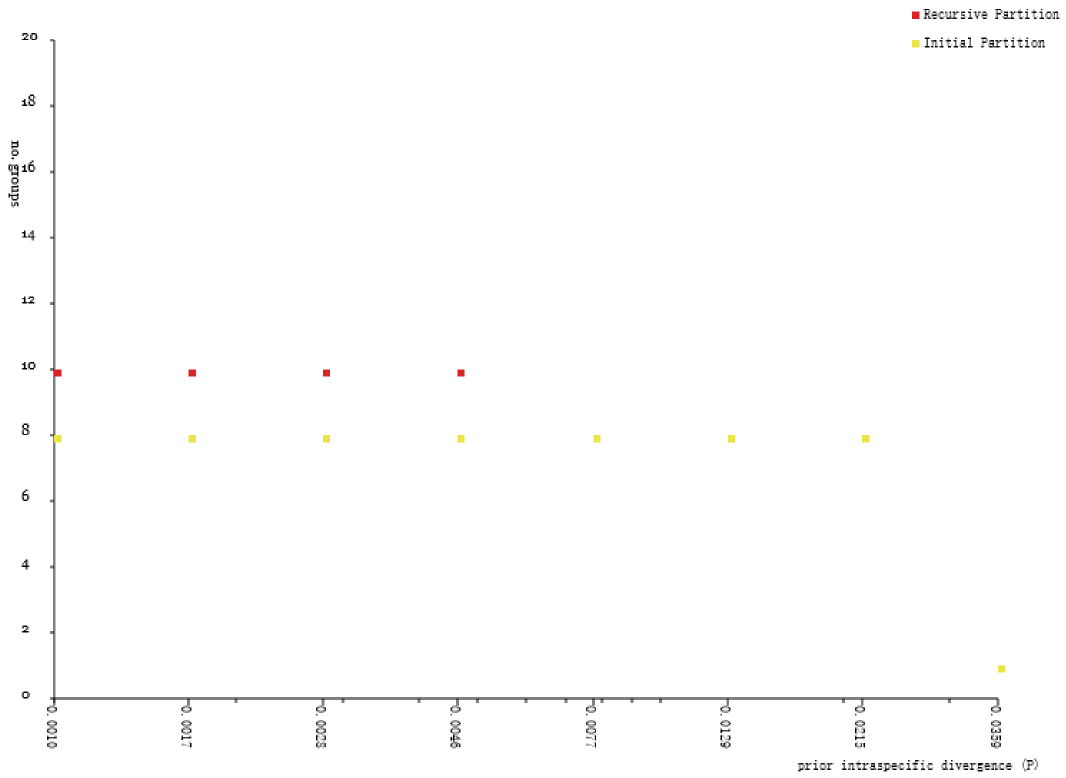

Supplementary Fig. 27. Automatic partition of tellinaceans based on *rbcL+tufA* sequences. The number of groups inside the partition (initial and recursive) of each given prior intraspecific divergence value were report.

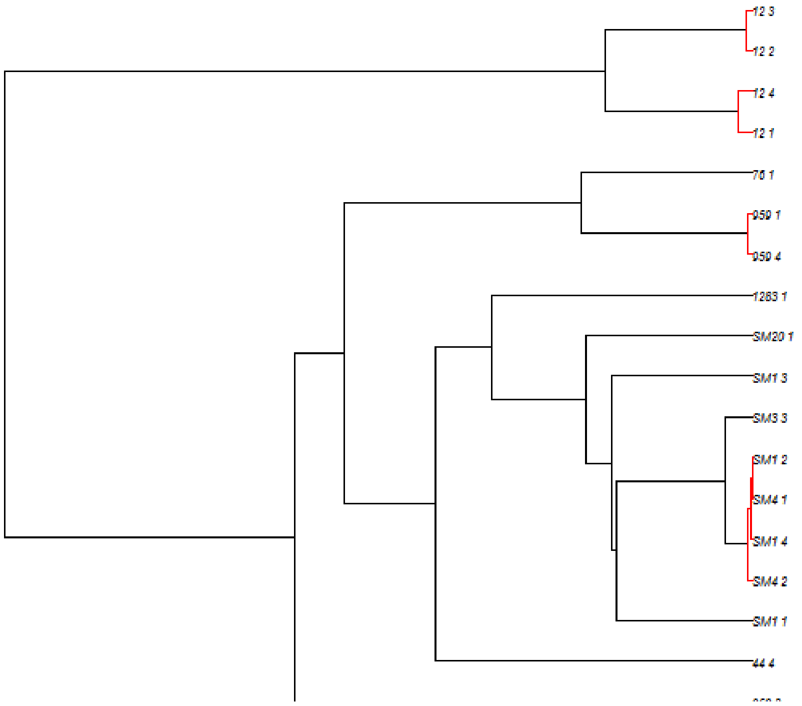

Supplementary Fig. 28. Genetic groups recovered by GMYC model for *rbcL+tufA*.

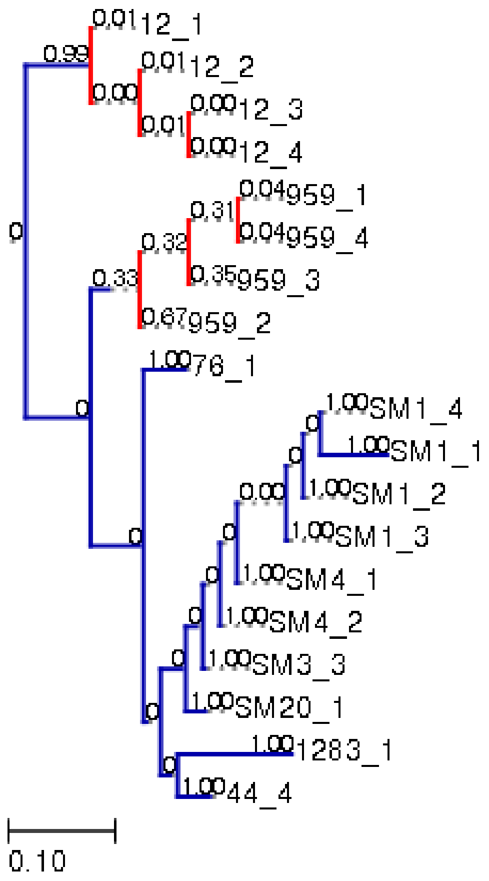

Supplementary Fig. 29. Genetic groups recovered by dPTP analysis for *rbcL+ tufA*.

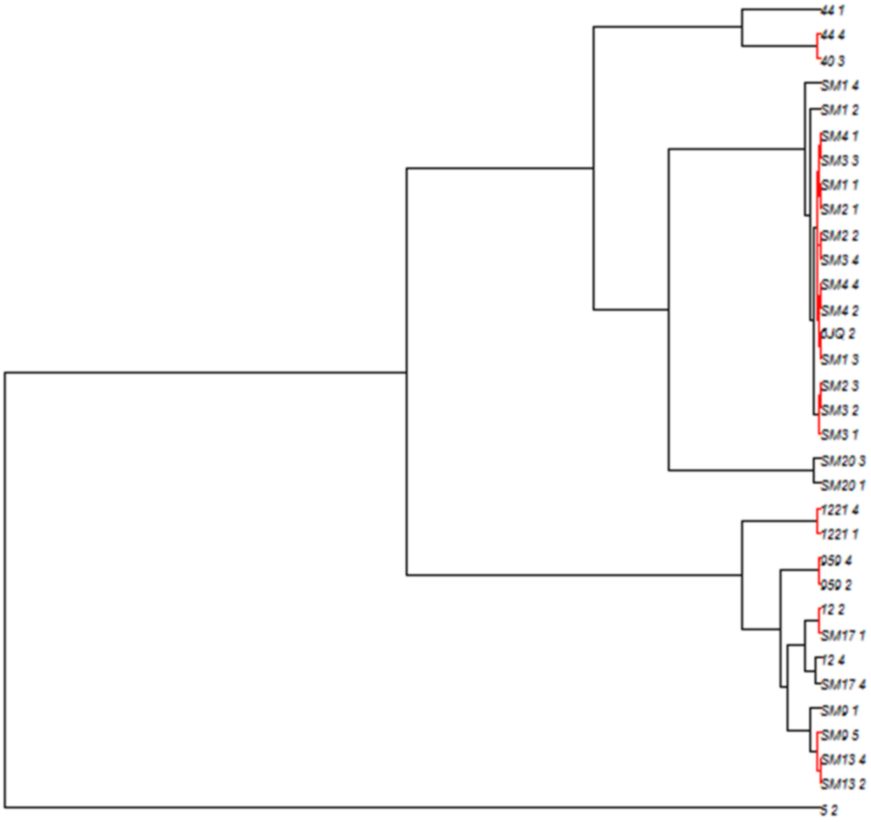

Supplementary Fig. 30. Genetic groups recovered by dPTP analysis for ITS+16S.

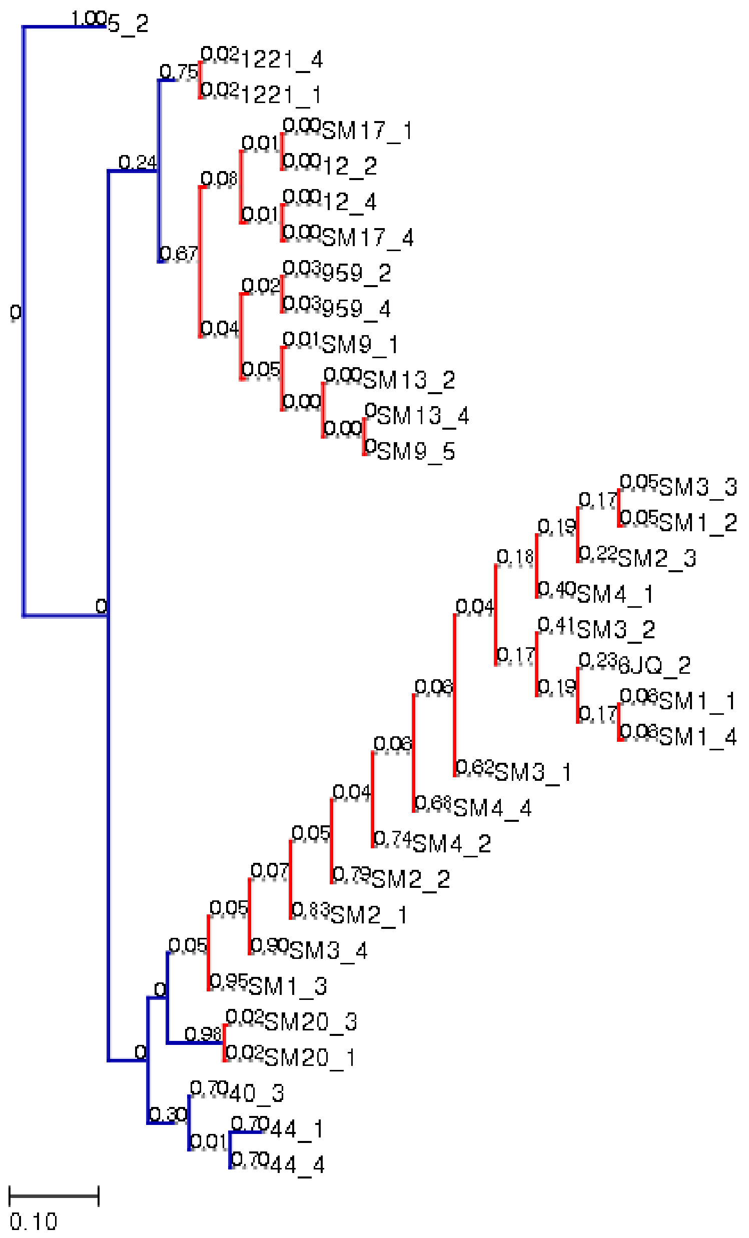

Supplementary Fig. 31. Genetic groups recovered by dPTP analysis for ITS+16S.
